# A scoping review of dietary assessment questionnaires potentially suitable for assessing habitual dietary intake in the National Health and Nutrition Survey, Japan

**DOI:** 10.1017/jns.2024.1

**Published:** 2024-02-12

**Authors:** Mai Matsumoto, Kentaro Murakami, Xiaoyi Yuan, Fumi Oono, Riho Adachi, Ryoko Tajima, Emiko Okada, Makiko Nakade, Satoshi Sasaki, Hidemi Takimoto

**Affiliations:** 1 Department of Nutritional Epidemiology and Shokuiku, National Institutes of Biomedical Innovation, Health, and Nutrition, Settsu-shi, Osaka, Japan; 2 Department of Social and Preventive Epidemiology, School of Public Health, The University of Tokyo, Bunkyo-ku, Tokyo, Japan; 3 Department of Social and Preventive Epidemiology, Division of Health Sciences and Nursing, Graduate School of Medicine, The University of Tokyo, Bunkyo-ku, Tokyo, Japan; 4 The Health Care Science Institute, Minato-ku, Tokyo, Japan; 5 Department of Food Science and Nutrition, University of Hyogo, Himeji, Hyogo, Japan; 6 Research Institute for Food and Nutritional Sciences, Himeji, Hyogo, Japan

**Keywords:** Dietary intake, FFQ, Japan, Nutrition survey, Questionnaire-based dietary assessment method

## Abstract

This scoping review aimed to identify questionnaire-based dietary assessment methods for use in the National Health and Nutrition Survey (NHNS) in Japan. The search was conducted in three databases (PubMed, Web of Science, and *Ichushi*) to identify questionnaire such as food frequency questionnaire and dietary history questionnaire validated against dietary recalls or food records for the intakes of both food groups and nutrients among Japanese adults. Study quality was assessed based on previously developed criteria. We extracted the questionnaire characteristics and the design and results of the validation studies. We identified 11 questionnaires, with the number of food items ranging from 40 to 196, from 32 articles of good quality. In the validation studies, participants were aged 30–76 years and 90% of the articles used ≥3 d dietary records as reference. The number of nutrients and food groups with a group-level intake difference within 20% against the reference method ranged from 1 to 30 and 1 to 11, respectively. The range of mean correlation coefficients between questionnaire and reference methods were 0.35–0.57 for nutrients and 0.28–0.52 for food groups. When selecting a survey instrument in the NHNS from the 11 existing questionnaires identified in this study, it is important to select one with high group-level comparison and correlation coefficient values on the intended assessment items after scrutinizing the design and results of the validation study. This review may serve as a reference for future studies that explore dietary assessment tools used for assessing dietary intake in specific representative populations.

## Introduction

The prevalence of non-communicable diseases (NCDs) such as diabetes and cardiovascular diseases has been increasing worldwide, and one of the factors reported to significantly influence their development is an unhealthy diet^(1,2)^. Therefore, it is necessary to improve dietary habits. Dietary intake at the population level has been regularly monitored in many countries using various dietary assessment methods, such as a dietary record, 24-h recall, and food frequency questionnaire (FFQ)^(3–8)^. The biggest challenge to a successful national nutrition survey is ensuring the representativeness of the sample population and accuracy of the collected data^(9,10)^.

The National Health and Nutrition Survey in Japan is a nationally representative cross-sectional annual survey for more than 70 years (with the exception of 2020 and 2021 because of the restrictions imposed during the COVID-19 pandemic), and conducted by local public health centres under the supervision of the Ministry of Health, Labour, and Welfare^(11)^. Details of the survey design have been described elsewhere^(11,12)^. Briefly, the NHNS consisted of a physical examination, a dietary survey, and a lifestyle questionnaire. Participants included households with family members aged ≥1 year in the 300 unit-blocks (approximately 5,700 households and 15,000 individuals) that were randomly selected from the unit-blocks of the Comprehensive Survey of Living Conditions, except for 2012 and 2016, when an expanded survey (approximately 23,750 households and 61,000 individuals) was conducted. However, the NHNS participants consist of more older adults (approximately 45%)^(11)^, and it has been questioned whether these results can be representativeness of the national population.

Although the NHNS assessed household dietary intake rather than individual intake using dietary record for 1–3 d until 1994, individual dietary intake has been assessed since 1995, using one-day semi-weighed household dietary records by applying the proportion of foods shared among family members to estimate individual intakes^(11)^. To explain in detail the current dietary record, the main recordkeepers (members who usually prepared meals in household) weighed and recorded not only all foods and beverages consumed by each household member but also the food waste and leftovers. Additionally, the main recordkeepers recorded the approximate proportions of food consumed by each household member when members shared foods. When family members are eating out and cannot measure their meals, the recordkeepers asked them for the portion size or quantity of foods consumed and any leftovers. These tasks are labor-intensive for recorders (especially those from multiple-member households and/or working age people) to report the dietary intake of each household member, as well as investigators who check the records. This is also one of the reasons why the participation rate in Japan is declining^(13)^, as evidenced in other countries^(14,15)^, and one solution would be to reduce the participant burden by shortening the time required to report dietary intake^(16,17)^. Thus, it is important to regularly evaluate and revise the dietary assessment methods used in national surveys to reduce respondent burden, thereby increasing participation and improving the accuracy of the estimation of dietary information^(18)^.

Another important issue is that, as mentioned above, the NHNS currently only assesses one-day dietary intake, making it unsuitable for use in comparing population habitual intake status with dietary reference intake (DRIs)^(19)^; for assessment of nutrient intake inadequacy. Developing assessment methods of habitual nutrients intake is needed which complements the current dietary assessment method of estimating daily intake with reduced participant burden^(20)^. Also, in many nutritional epidemiological studies, the relationship between dietary intakes and health outcomes has been investigated because diet is one of the major lifestyle-related risk factors^(21)^. However, it is not possible to assess the association between individual health status as measured by the NHNS Japan blood tests (complete blood count and blood biochemistry) and the intake of nutrients and foods assessed by NHNS, because it is inappropriate to assess the one-day dietary intake and health status^(11)^. Therefore, there is an urgent need for a review of dietary assessment methods that can serve as a complementary to the current one-day dietary record in the NHNS that can provide further evidence on the adequacy of nutrient intakes and can generate habitual intake to assess the relationship to health outcomes.

Questionnaire-based dietary assessment methods, such as FFQs and dietary history questionnaires, are widely used because they can be easily administered and assess habitual dietary intake over an extended period (a month or year)^(22)^. However, given that dietary habits vary among regions/countries and cultures^(23,24)^, FFQs and dietary history questionnaires that are appropriate for the target population should be used^(25)^. Several reviews have been conducted to identify dietary assessment questionnaires that can be used in epidemiological studies^(26–28)^; however, for dietary intake status, most studies have focused on the ability to rank individual dietary intakes (where they are located in the quartile). Only a few studies have focused on the ability to estimate intake at group level (group mean intake), which is frequently used to report the overall picture in national nutrition surveys^(11,29)^. Thus, it is essential to review the existing dietary questionnaires that can be used in the NHNS to assess the current Japanese diet, including intake at group level.

Previous reviews have summarised findings on the basis of reported articles rather than summarizing the characteristics of each questionnaire separately^(24,27,30,31)^, making it challenging to compare the features and utility of each questionnaire. This is because the medians or mean values of the statistics (e.g. correlation coefficients) may be influenced by the number of reported articles rather than by the characteristics of the questionnaire itself. A Scoping review is considered appropriate to identify the existence of validated FFQs and dietary history questionnaires that can be used in the NHNS.

In addition, it is extremely important to evaluate the quality of each article in review. To evaluate the quality of reviews on dietary assessment methods, the EURopean micronutrient RECommendations Aligned Network of Excellence (EURECCA), which was established as a tool for evaluating the quality of studies validating FFQ and adopted in review studies to evaluate the quality of the dietary intake validation studies^(28,30)^, is best suited^(32)^.

Further, reference methods for FFQ validation studies include studies using 24-h recalls and dietary records which are able to provide detailed dietary information, and biomarker-based studies. Validating questionnaires using biomarker studies are more objective and used as ‘gold standard’, but only a limited number of nutrients is available as recovery biomarkers^(23)^. On the one hand, 24-h recalls and dietary records are recommended as reference methods when examining the validity of the assessment of various nutrients using the FFQ and dietary history questionnaire at once^(23)^.

Therefore, the purpose of this scoping review was to identify dietary assessment questionnaires that have been validated against the 24-h recall or dietary record methods and can thus be used in the Japanese NHNS. This review may serve as a reference for future studies that also intent to explore dietary assessment tools used for assessing dietary intake among specific representative populations in other countries.

## Methods

The review protocol was drafted based on the methodology and guidance for the conduct of systematic scoping reviews^(33)^ and the Preferred Reporting Items for Systematic Reviews and Meta-Analyses (PRISMA) — Scoping Review Extension^(34)^.

### Data source and search strategy

A search was conducted to locate published studies from inception to May 31, 2022, using three databases (PubMed/Medline, Web of Science, and ‘Ichushi [Japanese database of health and medical science articles]’). The search was supplemented by a manual search of the reference lists of the included articles. To identify the FFQs or dietary history questionnaires that have been validated against a reference method (dietary record or 24-h recall methods) and that can assess dietary intake in the Japanese population, we included words related to ‘Japanese’, (‘FFQ’ or ‘dietary history questionnaire’), (‘dietary record’ or ‘24-h recall’), and ‘validation’. Detailed terms are shown in Supplementary Table 1.

### Inclusion and exclusion criteria

The inclusion criteria in this scoping review were manuscripts that 1) are peer-reviewed original research articles (full-text); 2) are published in English or Japanese; 3) describe studies conducted among the Japanese population; and 4) validate the daily intake of nutrients or food groups against a dietary record or 24-h recall.

We excluded the following references: 1) reviews, conference proceedings, and other non-original papers; 2) studies exclusively conducted among Japanese individuals living in foreign countries; 3) studies on animals; 4) no access to the full-text article; 5) studies involving only infants under 1 year of age (because the NHNS does not include participants under 1 year of age^(11)^); 6) studies involving only those who consume special diets, such as liquid diets; 7) studies assessing validity using only biomarkers; 8) studies in which the time interval between the conduct of questionnaires and reference methods exceeded 1 year; 9) studies examining the validity of specific nutrients or food groups (rather than the whole diet); 10) questionnaires assessing the intake of nutrients or food groups not reported in the NHNS; and 11) studies conducted among a specific population (e.g. students and parents attending a single school or university, athletes, only pregnant women in hospitals) rather than the general population.

### Selection of articles

After the search, all records retrieved from the relevant databases were exported to Microsoft Excel and duplicates were removed. The selection of articles was conducted in two steps. In Step 1, two researchers of the review team (M.M., X.Y., F.O., and R.A.) independently screened titles and abstracts for eligibility, and disagreements were resolved through consultation with a third reviewer (K.M.). Subsequently, the full texts of the articles extracted from the title and abstract screening were screened by a member of the review team, and one member’s perusal results were reviewed by another member of the review team. Additionally, the two researchers of the review team manually searched the reference lists of review articles to identify additional articles. Of the articles excluded from Step 1, those that evaluated validation of the nutrients or food groups not reported in the NHNS were re-evaluated and advanced to Step 2, even though they were found not to have been validated for the whole diet.

In Step 2, two members of the review team independently reviewed all articles and selected main articles according to the following three priorities: 1) studies involving healthy Japanese adults; 2) studies evaluating validity in terms of both nutrients and food groups reported in the NHNS in the same population (if the validity in terms of both nutrients and food groups was not evaluated in a single article, each article was considered as a main article); and 3) studies with the earliest publication date, if the validation of nutrients and food groups intakes was evaluated in multiple populations using the same method. In addition, articles other than those identified were included as additional articles (e.g. articles reporting validity in other populations, such as older adults or children in a unique life stage, and in terms of other nutrients). Disagreements were resolved through consultation with the third reviewer. The reason for implementing Step 2 is that some of the validation study articles evaluated several nutrients in a small group of people of some different ages from the first validation study article. If all these articles were included in the review, the number of validation study articles on the questionnaire could affect the evaluation. Therefore, we decided to classify main papers and additional articles according to the above criteria.

### Data extraction and synthesis

Independent data extraction from each article was conducted by a review team member using a standardized table (Excel sheet) specifically developed for synthesis in this review. The following data were extracted: 1) journal information including publication year and first author; 2) participant characteristics including sex, age, and sample size; 3) survey name (if any); 4) characteristics of the questionnaires including name (abbreviations, if any), reference period, tool type (paper or web-based), administration method (self-administered or interview), number of food items, and time required for completion; and 5) details of validation studies. In addition, for detail of validation studies, the following data were extracted: a) survey year; b) reference dietary assessment method; c) number of days’ intake assessed using the reference method; d) order of implementation of questionnaire and reference dietary assessment method; e) whether the reference method was properly conducted by nutrition experts (e.g. probing for additional or missing information after collection of dietary records); f) statistics employed to assess validity between questionnaires and reference methods for energy, nutrients, and food groups — including energy adjustment method in mean value calculation, type of correlation coefficient (CC) used (Pearson or Spearman), CC adjustment method (crude, adjusted such as for energy, or deattenuated or intraclass), CC value, and agreement (classification or Bland–Altman plot). Additionally, intake at the group level (mean or median) was extracted from only main articles.

For nutrients, we sorted our data according to the nutrients reported in the NHNS^(11)^; however, if additional articles reported on validity in terms of only nutrients not reported in the NHNS, they were also sorted. For food groups, we sorted the major categories of food groups (i.e. Grains; potato; sugar and sweeteners; beans; seeds and nuts; vegetables; fruits; mushrooms; seaweed; fish and shellfish; meat; egg; dairy; fat and oils; confectionaries; beverages; seasonings; and spices) reported in the NHNS, and food groups that may require monitoring because they are reported to have a significant effect on the development of NCDs and death, which include red meat, processed meat, and milk^(2)^. Additionally, as rice is a staple food of the Japanese population, accounting for approximately 75% of grain intake^(13)^, rice was added to the sorted list.

Using the extracted information, for the identified main articles, the percentage difference in mean or median intake for each questionnaire was calculated using the following formula:

(mean or median dietary intake calculated by questionnaires — mean or median dietary intake calculated by reference methods)/mean or median dietary intake calculated by reference methods × 100.

We also counted the number of nutrients within ± 20% of the difference in the mean or median intake in the main studies as per the criteria used in previous studies^(35,36)^. Furthermore, for the identified main articles, we calculated the means of the CCs for all nutrient (only reported in the NHNS) or food group intakes (reported in the NHNS or that have a significant influence on the global burden of disease and mortality^(2)^) for each questionnaire selected in this study.

### Quality assessment

All articles were independently evaluated for quality by two members of the review team. Study quality was assessed using a modified version of the EURRECA^(32)^, with some modifications to fit the purpose of this review. The reason for using the modified version for quality assessment in this review is that the original version was specific to dietary assessment of micronutrient intake^(32)^, whereas the present study covered all nutrients. The quality assessment included the following five domains: 1) sample and sample size; 2) statistics used to assess validity; 3) data collection; 4) seasonality; and 5) supplements.

For the first two domains, the original criteria were used: 1) sample (0.5 points when the sample was not homogeneous for characteristics such as sex and age) and sample size (0.5 points when the sample size exceeded 100) and 2) statistics used to assess validity for three items: a) comparison means, medians, and differences between two methods (1 point), b) correlation between two methods (crude [0.5 points], energy-adjusted [1 point], or deattenuated or intraclass [1.5 points]), and c) assessment for agreement or misclassification (0.5 points).

For data collection, the original criterion “face to face interview^(32)^ was further strengthened and scored in the following two categories: 1) the accuracy was set at 1 point when the content of dietary assessments was verified by a nutritionist or nutrition expert, because it has been reported that most questionnaires are designed to be self-administered and that the differences between self-administration and interview methods vary among nutrients^(23)^; 2) the order of implementation of questionnaires and reference dietary assessment methods was added as a criterion because it has been reported that a validity study can be conducted more accurately if the reference dietary assessment method is conducted after the questionnaire (0.5 points when the questionnaire was conducted before the reference dietary assessment method)^(23)^.

Seasonal difference in intake have been reported for certain nutrients and food groups, such as vitamin C and potato, but many nutrients have no significant effect when validity is examined over a single season as opposed to over an entire year^(37)^. Therefore, seasonal differences were not considered in this review, which focuses on nutrients as a whole, not just micronutrients. Moreover, in terms of the supplements domain, we decided not to adopt it as a criterion in the present study, because the nutrient content of supplements is not listed in the Japanese Food Composition Tables and it is difficult to accurately capture nutrient intake from supplements. The highest and lowest quality score was six and zero, respectively.

The quality of each study was considered as poor (< 2 points), acceptable (2 to < 3 points), good (3 to < 4 points), and excellent (≥ 4 points) in a modified version based on previously reported criteria^(32)^. If two or more questionnaires were validated using the same method (e.g. participants, reference dietary assessment method, etc.) within a single article, they were presented as results of a single article review.

## Results

### Study selection

**Fig. 1.**
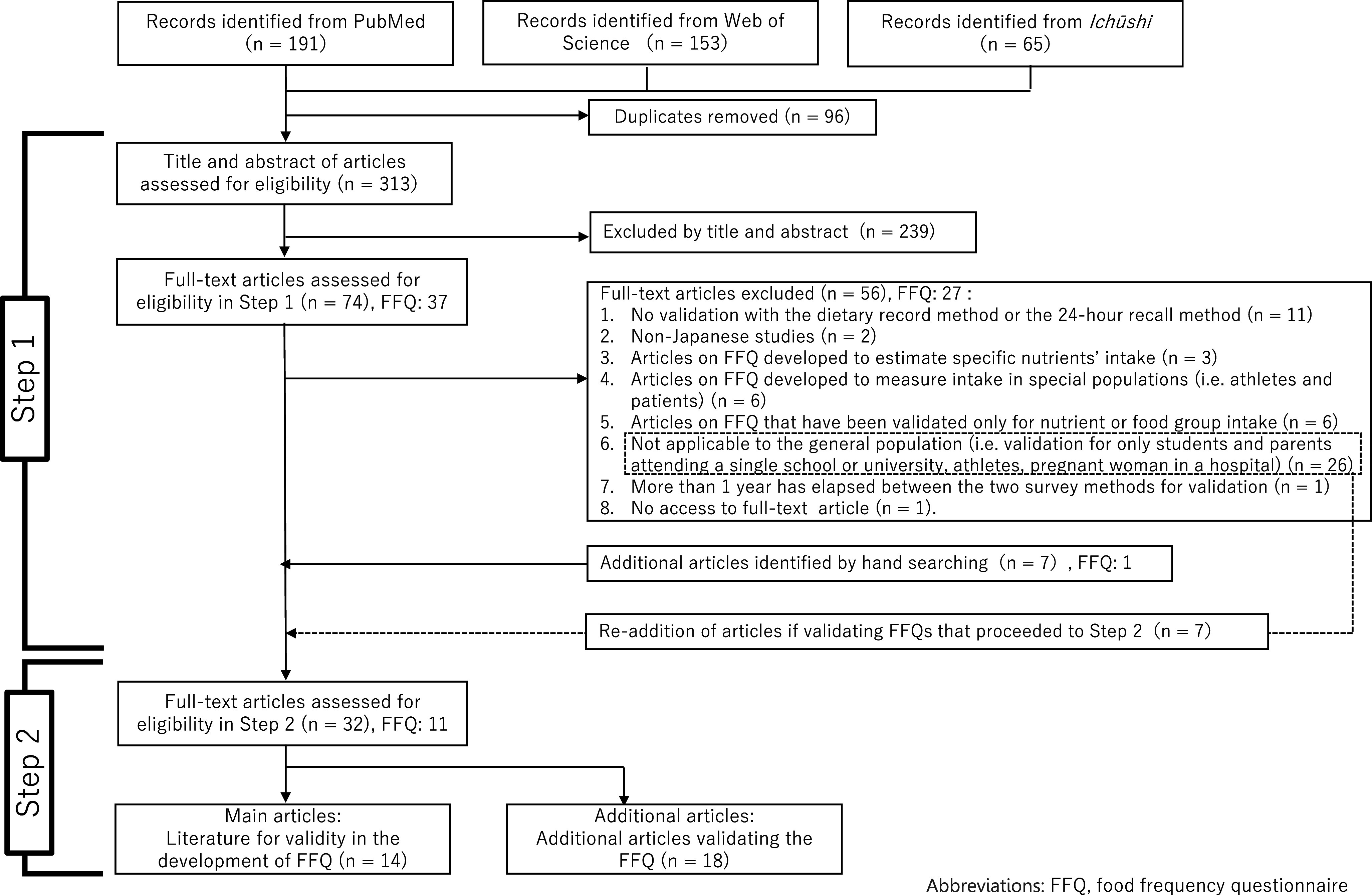
Flowchart of the study selection to identify questionnaire-based dietary assessment methods validated among Japanese adults for use in the National Health and Nutrition Survey. FFQ, food frequency questionnaire.

Figure 1 shows the results of the study selection. A total of 409 articles were extracted from three electronic databases. After excluding duplicates (n = 96) and articles which did not meet the inclusion criteria from the titles and abstracts (n = 239), 74 articles (37 questionnaires) were subjected to full-text reading (Steps 1 and 2). In Step 1, articles that did not meet the inclusion criteria were excluded (n = 56; 27 questionnaires); however, articles that validated the nine questionnaires that proceeded to Step 2 were re-added (n = 7) and additional articles that were found through hand searching were also added (n = 7, including another questionnaire). As a result, 32 articles^(38–69)^ which evaluated the validity of the following 11 questionnaires were included in this scoping review: ‘47-item short food frequency questionnaire (47-item FFQ)’^(38–41)^, ‘self-administered diet history questionnaire (DHQ)’^(42–45)^, ‘brief-type self-administered diet history questionnaire (BDHQ)’^(43–48)^, ‘Meal-based Diet History Questionnaire (MDHQ)’^(49–51)^, ‘FFQ in JACC’^(52,53)^, ‘44-item food frequency questionnaire (JPHC FFQ at baseline)^(54,55)^’, ‘JPHC FFQ at 5-year follow-up (JPHC_5y)’^(56–64)^, ‘66-item food frequency questionnaire for JPHC-NEXT follow-up survey short-FFQ (Short-FFQ JPHC-NEXT)’^(65)^, ‘food frequency questionnaire for JPHC-NEXT follow-up survey long-FFQ (FFQ in JPHC-NEXT)’^(65–67)^, ‘short version of the Shizuoka Prefecture version of the Food Intake Frequency Questionnaire (Short-version FFQ)’^(68)^ and ‘food frequency questionnaire (Maruyama FFQ)’^(69)^. In Steps 2, 14, and 18, articles were classified as main articles^(38,41,43,44,49–52,55–57,65,68,69)^ and additional articles^(39,40,42,45–48,53,54,58–64,66,67)^, respectively.

### Quality assessment

The results of the quality assessment are shown in Table 1. The range of total quality score of the 32 articles was 3–5.5 points (main articles: 3.5–5 points), with eight articles (including 2 main articles) classified as ‘good’, while the remaining articles were classified as ‘excellent’. The samples of four of the additional articles were classified as homogeneous. Moreover, the sample sizes were fewer than 100 participants in six articles. All but one of the selected references used multiple analysis methods, with correlation being the most frequently used statistical method (main [n = 14], additional [n = 17]) followed by group-level comparison (main [n = 14], additional [n = 16]). Seven main and nine additional articles administered questionnaires prior to the reference method.

**Table 1.** Study quality as assessed by modified EURECCA tool in a scoping review of validating dietary questionnaires used in Japan

FFQ	Year	First author	Total quality score	Quality	Sample: 1: non-homogeneous (0.5 points) 0: homogeneous	Sample size 1: >100 (0.5 points) 0: ≤100	Statistics&onecirc; Group-level: compare/test mean, median, or difference 1: Yes (1 point) 0: No	Statistics&twocirc; Correlations: 1: Correlation (0.5 points) 2: Adjusted correlation (energy) (1 point) 3: Deattenuated or intraclass correlation (1.5 points) 0: Unknown	Statistics&threecirc; Agreement: classification or Bland–Altman plot 1: Yes (0.5 points) 0: No	Data collection&onecirc; Nutritionist or someone familiar with dietary surveys confirmed content 1: Yes (1 point) 0: No or unknown	Data collection&twocirc; Order of implementation of FFQ and reference dietary assessment method 1: FFQ first (0.5 points) 0: Dietary assessment method first or unknown
47-item FFQ^(38)^	2005	Tokudome Y.	5.5	Excellent	1	1	1	3	1	1	1
47-item FFQ^(39)^	2016	Nakahata NT.	3	Good	1	0	0	3	1	0	1
47-item FFQ^(40)^	2019	Watanabe D.	4	Excellent	1	1	1	1	0	1	1
47-item FFQ^(41)^	2021	Imaeda N.	5.5	Excellent	1	1	1	3	1	1	1
DHQ^(42)^	1998	Sasaki S.	4.5	Excellent	0	0	1	3	1	1	1
DHQ, BDHQ^(43)^	2011	Kobayashi S.	5	Excellent	1	1	1	2	1	1	1
DHQ, BDHQ^(44)^	2012	Kobayashi S.	5	Excellent	1	1	1	3	0	1	1
DHQ, BDHQ^(45)^	2020	Fujiwara A.	5	Excellent	1	1	1	2	1	1	1
BDHQ^(46)^	2018	Saka H.	3.5	Good	1	1	1	0	1	1	0
BDHQ^(47)^	2019	Kobayashi S.	4.5	Excellent	1	0	1	2	1	1	1
BDHQ3y^(48)^	2015	Asakura K.	3.5	Good	0	0	1	2	0	1	1
MDHQ^(49)^	2022	Murakami K.	4.5	Excellent	1	1	1	1	1	1	1
MDHQ^(50)^	2022	Murakami K.	5	Excellent	1	1	1	2	1	1	1
MDHQ^(51)^	2022	Murakami K.	4	Excellent	1	1	1	1	0	1	1
FFQ in JACC^(52)^	2003	Ogawa K.	5	Excellent	1	1	1	3	1	1	0
FFQ in JACC^(53)^	2005	Date C.	4.5	Excellent	1	0	1	2	1	1	1
JPHC FFQ at baseline^(54)^	2001	Tsubono Y.	4	Good	1	1	0	3	0	1	1
JPHC FFQ at baseline^(55)^	2003	Tsubono Y.	4	Excellent	1	1	1	2	0	1	0
JPHC_5y^(56)^	2003	Tsugane S.	3.5	Good	1	1	1	2	1	0	0
JPHC_5y^(57)^	2003	Sasaki S.	3.5	Good	1	1	1	2	1	0	0
JPHC_5y^(58)^	2003	Karita K.	3	Good	1	1	1	2	0	0	0
JPHC_5y^(59)^	2003	Sasaki S.	3	Good	0	1	1	2	1	0	0
JPHC_5y^(60)^	2003	Ishihara J.	4	Excellent	1	1	1	2	0	1	0
JPHC_5y^(61)^	2005	Ishihara J.	4.5	Excellent	1	1	1	3	0	1	0
JPHC_5y^(62)^	2006	Ishihara J.	4	Excellent	1	1	1	2	0	1	0
JPHC_5y^(63)^	2011	Takachi R.	5.5	Excellent	1	1	1	3	1	1	1
JPHC_5y^(64)^	2021	Mori N.	4.5	Excellent	1	1	1	3	0	1	0
Short-FFQ in JPHC-NEXT, FFQ in JPHC-NEXT^(65)^	2016	Yokoyama Y.	5	Excellent	1	1	1	3	1	1	0
FFQ in JPHC-NEXT^(66)^	2016	Sunami A.	4.5	Excellent	0	1	1	3	1	1	0
FFQ in JPHC-NEXT^(67)^	2017	Kato E.	5	Excellent	1	1	1	3	1	1	0
Short version FFQ^(68)^	2015	Akahori M.	4	Excellent	1	1	1	2	0	1	0
Maruyama FFQ^(69)^	2015	Maruyama K.	4	Excellent	1	0	1	2	1	1	0

FFQ, Food frequency questionnaire; 47-item FFQ, 47-item short food frequency questionnaire; DHQ, Self-administered diet history questionnaire; BDHQ, Brief-type self-administered diet history questionnaire; MDHQ, Meal-based Diet History Questionnaire; JPHC FFQ at baseline, 44-item food frequency questionnaire; JPHC_5y, JPHC FFQ at 5-year follow-up; FFQ in JPHC-NEXT, Long-FFQ in the Japan Public Health Centre-based prospective Study for the Next Generation (JPHC-NEXT); Short-FFQ in JPHC-NEXT, 66-item food frequency questionnaire for the Japan Public Health Centre-based prospective Study for the Next Generation (JPHC-NEXT) follow-up survey; Short version FFQ, Short version of the Shizuoka Prefecture version of the Food Intake Frequency Questionnaire; Maruyama FFQ, Maruyama food frequency questionnaire

### Questionnaire characteristics

Table 2 shows the characteristics of the 11 questionnaires (32 validation study articles) identified in this scoping review. All questionnaires were self-administered. The time periods measured by FFQ was 1 month for five of the questionnaires (DHQ, BDHQ, MDHQ, JPHC FFQ at baseline, and Short-version FFQ) and 1 year for four questionnaires (47-item FFQ, JPHC_5y, FFQ in JPHC-NEXT, and Short-FFQ in JPHC-NEXT), while two questionnaires (FFQ in JACC and Maruyama FFQ) did not state the time periods. All questionnaires were available in paper form, with two of them also available in electronic form (MDHQ and FFQ in JPHC-NEXT). The number of food items ranged from 40 (FFQ in JACC) to 196 (MDHQ). Only three questionnaires (DHQ: 45–60 min, BDHQ: 15–20 min, MDHQ: < 20 min for approximately 65% population) indicated the time taken to complete (15–60 min).

**Table 2.** Characteristics of included validation studies in a scoping review of dietary assessment questionnaires used in Japan; survey administration

FFQ	Time periods measured by FFQ	Tool 1: Paper 2: Electronic	Administration method 1: Self-administered 2: Interview	Number of food items included in the questionnaire	Time taken to complete (minutes)	Notes
47-item FFQ	1 year	1	1	47	Not Stated	
DHQ	1 month	1	1	150	45-60	
BDHQ	1 month	1	1	58	15-20	
MDHQ	1 month	1 and 2	1	196	Less than 20 min (63.5%) 20 min or more (36.5%)	To estimate dietary intake for each meal type (breakfast, morning snack, lunch, afternoon snack, dinner, and night snack)
FFQ in JACC	N/A	1	1	40	Not Stated	
JPHC FFQ at baseline	1 month	1	1	44	Not Stated	
JPHC_5y	1 year	1	1	138	Not Stated	
FFQ in JPHC-NEXT	1 year	1 and 2	1	172	Not Stated	
Short-FFQ in JPHC-NEXT	1 year	1	1	66	Not Stated	
Short version FFQ	1 month	1	1	86	Not Stated	
Maruyama FFQ	N/A	1	1	81	Not Stated	

FFQ, Food frequency questionnaire; 47-item FFQ, 47-item short food frequency questionnaire; DHQ, Self-administered diet history questionnaire; BDHQ, Brief-type self-administered diet history questionnaire; MDHQ, Meal-based Diet History Questionnaire; JPHC FFQ at baseline, 44-item food frequency questionnaire; JPHC_5y, JPHC FFQ at 5-year follow-up; FFQ in JPHC-NEXT, Long-FFQ in the Japan Public Health Centre-based prospective Study for the Next Generation (JPHC-NEXT); Short-FFQ in JPHC-NEXT, 66-item food frequency questionnaire for the Japan Public Health Centre-based prospective Study for the Next Generation (JPHC-NEXT) follow-up survey; Short version FFQ, Short version of the Shizuoka Prefecture version of the Food Intake Frequency Questionnaire; Maruyama FFQ, Maruyama food frequency questionnaire

### Characteristics of the validity studies for questionnaires

Table 3 shows the characteristics of the validity studies for questionnaires. Participants in the main papers ranged in age from 30 to 76 years, most studies incorporate a subset of participants representing the middle years of 40 to 75 years old participating in the cohort study in which the FFQs are used. This trend was similar in the additional articles, although the 47-item FFQ, DHQ, BDHQ, MDHQ, and FFQ in JACC included young adults (<40 years), the 47-item FFQ and BDHQ included older adults (≥ 80 years), and the BDHQ included young children (3–4 years). Additionally, the number of prefectures from which participants were sampled for validity studies (indicated by the minimum value if there were several studies) ranged from 1 prefecture (Short version FFQ and Maruyama FFQ) to 14 prefectures (MDHQ). In all the studies, the reference dietary assessment method was the dietary record method, except for an additional article using the 24-h recall method. The number of days of intake assessed using the reference methods ranged from 1 to 28 d. Approximately 90% of the articles used 3 or more days dietary records as reference, and one-day dietary record was used as a reference method in two articles.

**Table 3.** Characteristics of included validation studies in a scoping review of dietary assessment questionnaires used in Japan; study details

Year	First author	FFQ	Group M: main report A: Additional report	Participant characteristics in validation study						Survey year	Reference dietary assessment method	Number of reference method collection days	Validation for energy intake 1: Yes 0: No	Validation for nutrient intake 1: Yes 0: No	Validation for food group intake 1: Yes 0: No
				Sex M: Male F: Female	Age (years); range	Survey name (including only a subsample of the participants)	Survey area (prefectures)s	Other characteristics	Sample size						
2005	Tokudome Y.	47-item FFQ^(38)^	M	M and F	30–70	N/A	Aichi Pref.	People who attended physical exercise classes in communities, or who were parents of college students in Aichi Prefecture, Central Japan	202	2004	Dietary records	3	1	1	0
2016	Nakahata NT.	47-item FFQ^(39)^	A	M and F	40–69	Residents of the Amami Islands area	Amami Islands (Okinawa)	Men and women of married couples	66	2009	Dietary records	3 or 12	1	1	0
2019	Watanabe D.	47-item FFQ^(40)^	A	M and F	Male: 66–88 Female: 65–85	The Kyoto–Kameoka Study	Kamaoka City (Kyoto)	N/A	143	2012	Dietary records	7	1	1	0
2021	Imaeda N.	47-item FFQ^(41)^	M	M and F	35–69	Japan Multi-Institutional Collaborative Cohort Study (J-MICC study)	11 areas (8 prefs.): Yamagata, Tsuruoka, Chiba, Shizuoka–Sakuragaoka, Shizuoka, Okazaki, Aichi, Takashima, Kyoto, Tokushima, Saga)	The participants in the J-MICC study	288	2011–2013	Dietary records	12	0	0	1
1998	Sasaki S.	DHQ^(42)^	A	F	38–69	N/A	Hakone City	Women with mild hypercholesterolemia living in Hikone, Japan	47	1995	Dietary records	3	1	1	0
2011	Kobayashi S.	DHQ, BDHQ^(43)^	M	M and F	Male: 32–76 Female: 31–69	N/A	3 areas: Osaka Pref., Nagano Pref., Tottori Pref.	Men and women of married couples in Osaka (urban area), Nagano (rural inland area), and Tottori (rural coastal area) Prefectures	184 Male: 92 Female: 92	2002–2003	Dietary records	16	1	0	1
2012	Kobayashi S.	DHQ, BDHQ^(44)^	M	M and F	Male: 32–76 Female: 31–69	N/A	3 areas: Osaka Pref., Nagano Pref., Tottori Pref.	Men and women of married couples in Osaka (urban area), Nagano (rural inland area), and Tottori (rural coastal area) Prefectures	184 Male: 92 Female: 92	2002–2003	Dietary records	16	1	1	0
2020	Fujiwara A.	DHQ, BDHQ^(45)^	A	M and F	31–76	N/A	3 areas: Osaka Pref., Nagano Pref., Tottori Pref.	Men and women of married couples	184 Male: 92 Female: 92	2002–2003	Dietary records	16	0	1	0
2018	Saka H.	BDHQ^(46)^	A	M and F	40–69	National Health and Nutrition Survey	Shimane Pref.	N/A	343	N/A	Dietary records	1	1	1	1
2019	Kobayashi S.	BDHQ^(47)^	A	M and F	82–94	The TOOTH study; The SONIC study	Tokyo Pref.	N/A	80 Male: 36 Female: 44	2012–2015	Dietary records	3	1	1	1
2015	Asakura K.	BDHQ3y^(48)^	A	M and F	3–4	N/A	Tome City (Miyagi)	Children	61	2008–2009	Dietary records	3	1	1	1
2022	Murakami K	MDHQ^(49)^	M	M and F	30–76	N/A	14 prefs.	Men and women of married couples in 14 (of the 47) prefectures	222 Male: 111 Female: 111	2021	Dietary records	4	0	0	1
2022	Murakami K	MDHQ^(50)^	M	M and F	30–76	N/A	14 prefs.	Men and women of married couples in 14 (of the 47) prefectures	222 Male: 111 Female: 111	2021	Dietary records	4	0	1	0
2022	Murakami K	MDHQ^(51)^	M	M and F	30–76	N/A	14 prefs.	Men and women of married couples in 14 (of the 47) prefectures	222 Male: 111 Female: 111	2021	Dietary records	4	1	0	0
2003	Ogawa K.	FFQ in JACC^(52)^	M	M and F	Male: 45–77 Female: 47–76	JACC study	2 rural towns (Miyagi)	Participants in the cohort study	113 Male: 55 Female: 58	1996–1997	Dietary records	12	1	1	1
2005	Date C.	FFQ in JACC^(53)^	A	M and F	20–79	JACC study	11 areas (8 prefs.): Yakumo Town (Hokkaido), Omori Town (Akita), Samukawa Town (Kanagawa), Higashi–Yamanashi County (Yamanashi), Shirakawa Town and Gifu City (Gifu), Wachi, Keihoku and Wazuka Towns (Kyoto), Ichinomiya Town (Hyogo), Saikawa Town (Fukuoka)	A subsample of participants in the JACC study	85	1997–1999	Dietary records	12	1	1	0
2001	Tsubono Y.	JPHC FFQ at baseline^(54)^	A	M and F	40–59	JPHC study	4 areas: Nihohe, Yokote, Saku, Ishikawa	The participants in the JPHC cohort I study	201 Male: 94 Female: 107	1990–1995	Dietary records	3	0	1	0
2003	Tsubono Y.	JPHC FFQ at baseline^(55)^	M	M and F	40–59	JPHC study	4 areas: Nihohe, Yokote, Saku, Ishikawa	A subsample of participants in the JPHC cohort I study	201	1994	Dietary records	28 or 14	1	1	1
2003	Tsugane S.	JPHC_5y^(56)^	M	M and F	N/A	JPHC study	4 areas: Iwate, Akita, Nagano, Okinawa	The participants in the JPHC study	215	N/A	Dietary records	28 or 14	1	1	0
2003	Sasaki S.	JPHC_5y^(57)^	M	M and F	N/A	JPHC study	4 areas: Iwate, Akita, Nagano, Okinawa	The participants in the JPHC study	215	N/A	Dietary records	28 or 14	0	0	1
2003	Karita K.	JPHC_5y^(58)^	A	M and F	N/A	JPHC study	4 areas: Ninohe (Iwate Pref.), Yokote (Akita Pref.), Saku (Nagano Pref.), Ishikawa (Okinawa Pref.)	The participants in the JPHC study	215 Male: 102 Female: 113	N/A	Dietary records	28 or 14	0	1	0
2003	Sasaki S.	JPHC_5y^(59)^	A	M and F	N/A	N/A	N/A	N/A	215	N/A	Dietary records	28 or 14	0	1	1
2003	Ishihara J.	JPHC_5y^(60)^	A	M and F	40–69	JPHC study	14 areas (6 prefs.): Oguni Town (Niigata); Iwase and Tomobe Towns (Ibaraki); Suita City (Osaka); Noichi and Kagami Towns (Kochi Pref.); Uku, Odika, Shin-uonome, Arikawa, Kamigoto, and Narao Towns (Nagasaki Pref.), Gusukube and Hirara Cities (Okinawa Pref.)	A subsample of participants in the JPHC cohort II study	392	1996–1998	Dietary records	28	1	1	1
2005	Ishihara J.	JPHC_5y^(61)^	A	M and F	N/A	JPHC study	11 areas (10 prefs.): Yokote (Akita), Ninohe (Iwate), Kashiwazaki (Niigata), Mito (Ibaraki), Saku (Nagano), Katsushika (Tokyo), Suita (Osaka), Kamigoto (Nagasaki), Chubu and Miyako (Okinawa)	A subsample of participants in the JPHC study	565	1994–1998	Dietary records	28	1	1	0
2006	Ishihara J.	JPHC_5y^(62)^	A	M and F	45–74	JPHC-cohort I and JPHC-cohort II	11 areas (10 prefs.): Yokote (Akita), Ninohe (Iwate), Kashiwazaki (Niigata), Mito (Ibaraki), Saku (Nagano), Katsushika (Tokyo), Suita (Osaka), Kamigoto (Nagasaki), Chubu and Miyako (Okinawa)	JPHC participants, assumed to be married couples	565 JPHC-cohort I Male: 102 Female: 113 JPHC-cohort II Male: 174 Female: 176	1994–1998	Dietary records	28	1	1	0
2011	Takachi R.	JPHC_5y^(63)^	A	M and F	40–69	JPHC study	Tokyo Pref.	Visitors to the National Cancer Centre for Cancer Prevention and Screening between 2004 and 2006	143	2007–2008	Dietary records	4	1	1	1
2021	Mori N.	JPHC_5y^(64)^	A	M and F	Cohort I 40–59 Cohort II 40–69	JPHC-cohort I and JPHC-cohort II	11 areas (10 prefs.): Yokote (Akita), Ninohe (Iwate), Kashiwazaki (Niigata), Mito (Ibaraki), Saku (Nagano), Katsushika (Tokyo), Suita (Osaka), Kamigoto (Nagasaki), Chubu and Miyako (Okinawa)	A subsample of participants in the JPHC cohort I and II study	JPHC-cohort I: 209 JPHC-cohort II: 289	1990s	Dietary records	28	0	1	0
2016	Yokoyama Y.	Short-FFQ in JPHC-NEXT, FFQ in JPHC-NEXT^(65)^	M	M and F	40–74	JPHC-NEXT study	5 areas (4 prefs.): Yokote (Akita), Saku (Nagano), Chikusei (Ibaraki), Murakami and Uonuma (Niigata)	The participants in the JPHC-NEXT study	240	2012–2013	Dietary records	12	1	1	1
2016	Sunami A.	FFQ in JPHC-NEXT^(66)^	A	M and F	N/A	N/A	Tokyo Pref.	University student athletes	156	2013	24 h-recall	3	1	1	1
2017	Kato E.	FFQ in JPHC-NEXT^(67)^	A	M and F	40–74	JPHC-NEXT study	5 areas (4 prefs.): Yokote (Akita), Saku (Nagano), Chikusei (Ibaraki), Murakami and Uonuma (Niigata)	The participants in the JPHC-NEXT study	237	2012–2013	Dietary records	12	1	1	1
2015	Akahori M.	Short version FFQ^(68)^	M	M and F	Mean: 54.8	National Health and Nutrition Survey	Shizuoka Pref.	The participants in the 2008 Comprehensive Survey of Living Conditions	491	2008–2009	Dietary records	1	1	1	1
2015	Maruyama K.	Maruyama FFQ^(69)^	M	M and F	47–78	Suita study	Suita City	The participants in the validation study of the JPHC cohort II study who live in urban areas	58	1997–1998	Dietary records	28	1	1	1

FFQ, Food frequency questionnaire; 47-item FFQ, 47-item short food frequency questionnaire; DHQ, Self-administered diet history questionnaire; BDHQ, Brief-type self-administered diet history questionnaire; MDHQ, Meal-based Diet History Questionnaire; JPHC FFQ at baseline, 44-item food frequency questionnaire; JPHC_5y, JPHC FFQ at 5-year follow-up; FFQ in JPHC-NEXT, Long-FFQ in the Japan Public Health Centre-based prospective Study for the Next Generation (JPHC-NEXT); Short-FFQ in JPHC-NEXT, 66-item food frequency questionnaire for the Japan Public Health Centre-based prospective Study for the Next Generation (JPHC-NEXT) follow-up survey; Short version FFQ, Short version of the Shizuoka Prefecture version of the Food Intake Frequency Questionnaire; Maruyama FFQ, Maruyama food frequency questionnaire

### Outcomes of the validity studies

Tables 4 and 5 show the results of the evaluation of validity in terms of nutrient and food group intake in the main articles, respectively. For details of the validity of the FFQ, the results in terms of nutrient and food group intake are shown in Supplementary Tables 2 and 3, respectively. For nutrients, the number of nutrients reported in the NHNS that were validated in each questionnaire ranged from 12 (FFQ in JACC) to 32 (MDHQ). Among these nutrients, the number of group-level comparisons within 20% against reference methods ranged from 1 (FFQ in JACC) to 30 (DHQ and FFQ in JPHC-NEXT), and the mean proportion of nutrients with a group-level comparison within 20% was 58%. The highest number of group-level comparisons exceeding 20% against reference methods was 27 nutrients in Short-FFQ in JPHC-NEXT. Carbohydrates consistently had a high agreement in group-level comparison against reference methods using all 11 questionnaires, with mean differences in group-level comparison ranging from −22% to 11%.

**Table 4.** The results of the validity assessment of dietary questionnaires to estimate nutrient intakes among Japanese adults in main articles

FFQ	Participant characteristics			Energy-adjustment methods in mean value calculation 1: Crude 2: Density method 3: Residual method	Statistics: group-level intake 1. Mean 2. Median	Statistics: Correlations 1: Correlation 2: Adjusted correlation (energy) 3: deattenuated or intraclass correlation	Type of correlation coefficient between the FFQ and DR P: Pearson S: Spearman	Energy	
								Group level: Difference (%): (FFQ−DR)/DR*100	Correlation coefficient between the FFQ and DR
	Sex M: Male F: Female	Age (years)	n						
47-item FFQ^(38)^	M	30–68	73	1	1	3	P	−15	0.49
	F	30–68	129	1	1	3	P	−15	0.44
DHQ^(44)^	M	32–76	92	3	1	3	P	−5	0.42
	F	31–69	92	3	1	3	P	2	0.32
BDHQ^(44)^	M	32–76	92	3	1	3	P	−9	0.24
	F	31–69	92	3	1	3	P	−7	0.31
MDHQ^(51)^	M	30–76	111	2	1	2	P	–	–
	F	30–69	111	2	1	2	P	–	–
FFQ in JACC^(52)^	M	45–77	55	1	1	3	S	−16	0.55
	F	47–76	58	1	1	3	S	−27	0.36
JPHC FFQ at baseline^(55)^	M	40–59	94	3	1	2	S	−15	0.52
	F	40–59	107	3	1	2	S	−25	0.38
JPHC_5y^(56)^	M	–	102	3	1	2	S	0	0.55
	F	–	113	3	1	2	S	11	0.44
FFQ in JPHC-NEXT^(65)^	M	40–74	98	1	1	3	S	3	0.45
	F	40–74	142	1	1	3	S	13	0.17
Short-FFQ in JPHC-NEXT^(65)^	M	40–74	92	1	1	3	S	−21	0.49
	F	40–74	136	1	1	3	S	−24	0.16
Short version FFQ^(68)^	MF	Mean: 55.7	491	3	1	2	P	7	–
Maruyama FFQ^(69)^	MF	47–78	58	3	1	2	S	−7	–

FFQ	Nutrients											
	Number of validated nutrients	Nutrients reported in the NHNS										
		Number of validated nutrients	Difference (%): (FFQ−DR)/DR*100^†^					Correlation coefficient between the FFQ and DR				
			Number of nutrients with a difference between FFQ and DR within 20%	Low		High		Mean	Low		High	
				Nutrient	Value	Nutrient	Value		Nutrient	Value	Nutrient	Value
47-item FFQ^(38)^	23	20	7	Vitamin A	6	Dietary fibre, soluble	−43	0.44	n-6 polyunsaturated fatty acids	0.12	Carbohydrates	0.86
	23	20	13	Vitamin A	−1	Dietary fibre, soluble	−39	0.38	Vitamin B_1_	0.10	Carbohydrates	0.64
DHQ^(44)^	41	30	27	Vitamin K	−1	Folate	−23	0.52	Vitamin A	0.23	Dietary fibre, total and insoluble	0.73
	41	30	30	Monounsaturated fatty acids	2	Vitamin K	14	0.57	n-3 polyunsaturated fatty acids	0.36	Saturated fatty acids	0.75
BDHQ^(44)^	41	30	27	Cholesterol	0	Vitamin K	28	0.54	n-3 polyunsaturated fatty acids Niacin	0.25	Dietary fibre, total	0.72
	41	30	27	n-3 polyunsaturated fatty acids	0	Vitamin C	35	0.56	n-3 polyunsaturated fatty acids Cholesterol	0.34	Saturated fatty acids	0.70
MDHQ^(51)^	46	32	7	Sodium	−9	Vitamin A	−47	0.42	Niacin	0.15	Carbohydrates	0.66
	46	32	22	Sodium	−4	Vitamin B_12_	−31	0.41	n-6 polyunsaturated fatty acids	0.27	Carbohydrates	0.67
FFQ in JACC^(52)^	14	12	1	Carbohydrates	−6	Sodium	−59	0.43	Protein	0.25	Vitamin C	0.58
	14	12	1	Vitamin C	−17	Sodium	−56	0.48	Vitamin B_1_	0.31	Phosphorus	0.69
JPHC FFQ at baseline^(55)^	30	19	4	Carbohydrates	−2	n-3 polyunsaturated fatty acids	−63	0.35	n-6 polyunsaturated fatty acids Niacin	0.14	Calcium Phosphorus	0.56
	30	19	5	Calcium	−5	n-3 polyunsaturated fatty acids	−60	0.35	Niacin	0.11	Saturated fatty acids	0.50
JPHC_5y^(56)^	16	13	11	Phosphorus	1	Vitamin C	29	0.41	Protein	0.30	Carbohydrates	0.56
	16	13	10	Carbohydrates	7	Vitamin C	40	0.36	Niacin	0.15	Sodium	0.48
FFQ in JPHC-NEXT^(65)^	49	31	30	Monounsaturated fatty acids	−1	Vitamin B_12_	−21	0.47	Niacin	0.28	Carbohydrates	0.74
	49	31	22	Vitamin B_12_	3	Vitamin A	39	0.44	Monounsaturated fatty acids	0.21	Vitamin C	0.66
Short-FFQ in JPHC-NEXT^(65)^	48	31	4	Vitamin A	6	Vitamin C	−55	0.46	Vitamin B_12_	0.28	Carbohydrates	0.68
	48	31	10	Niacin	−2	Vitamin C	−40	0.44	Niacin	0.18	Copper	0.65
Short version FFQ^(68)^	32	29	27	Vitamin E	0	Niacin	85	0.37	Cholesterol	0.14	Vitamin C	0.55
Maruyama FFQ^(69)^	32	25	19	Animal protein	−1	Vitamin C	−37	0.48	n-3 polyunsaturated fatty acids	0.15	Carbohydrates	0.76

FFQ, Food frequency questionnaire; DR, dietary record; NHNS, National Health and Nutrition Survey; 47-item FFQ, 47-item short food frequency questionnaire; DHQ, Self-administered diet history questionnaire; BDHQ, Brief-type self-administered diet history questionnaire; MDHQ, Meal-based Diet History Questionnaire; JPHC FFQ at baseline, 44-item food frequency questionnaire; JPHC_5y, JPHC FFQ at 5-year follow-up; FFQ in the Japan Public Health Centre-based prospective Study for the Next Generation (JPHC-NEXT), Long-FFQ in JPHC-NEXT; Short-FFQ in JPHC-NEXT, 66-item food frequency questionnaire for the Japan Public Health Centre-based prospective Study for the Next Generation (JPHC-NEXT) follow-up survey; Short version FFQ, Short version of the Shizuoka Prefecture version of the Food Intake Frequency Questionnaire; Maruyama FFQ, Maruyama food frequency questionnaire

† Differences reported in absolute values

**Table 5.** The results of the validity assessments of dietary questionnaires to estimate food group intakes among Japanese adults in main articles

FFQ	Participant characteristic			Energy-adjustment methods in mean value calculation 1: Crude 2: Density method 3: Residual method	Statistics: group-level intake 1. Mean 2. Median	Statistics: Correlations 1: Correlation 2: Adjusted correlation (energy) 3: deattenuated or intraclass correlation	Type of correlation coefficient between the FFQ and DR P: Pearson S: Spearman	Energy	
	Sex M: Male F: Female	Age (years)	*n*					Group level: Difference (%): (FFQ−DR)/DR*100	Correlation coefficient between the FFQ and DR
47-item FFQ^(41)^	M	35–69	143	3	1	–	–	2	–
	F	35–69	145	3	1	–	–	11	–
DHQ^(43)^	M	32–76	92	2	2	2	S	–	–
	F	31–69	92	2	2	2	S	–	–
BDHQ^(43)^	M	32–76	92	2	2	2	S	–	–
	F	31–69	92	2	2	2	S	–	–
MDHQ^(49)^	M	30–76	111	1	2	1	S	–	–
	F	30–69	111	1	2	1	S	–	–
FFQ in JACC^(52)^	M	45–77	55	1	1	3	S	−16	0.55
	F	47–76	58	1	1	3	S	−27	0.36
JPHC FFQ at baseline^(55)^	M	40–59	94	3	1	2	S	−15	0.52
	F	40–59	107	3	1	2	S	−25	0.38
JPHC_5y^(57)^	M	–	102	3	1	2	S	–	–
	F	–	113	3	1	2	S	–	–
FFQ in JPHC-NEXT^(65)^	M	40–74	98	1	1	3	S	3	0.45
	F	40–74	142	1	1	3	S	13	0.17
Short-FFQ in JPHC-NEXT^(65)^	M	40–74	92	1	1	3	S	−21	0.49
	F	40–74	136	1	1	3	S	−24	0.16
Short version FFQ^(68)^	MF	Mean: 55.7	491	2	1	2	S	7	–
Maruyama FFQ^(69)^	MF	47–78	58	3	1	2	S	−7	–

FFQ	Food groups												
	Number of validated foods	Number of validated foods reported in the NHNS	Number of validated foods reported as main groups in the NHNS or as dietary risk factors for mortality and the GBD	Food groups reported in the NHNS or as dietary risk factors for mortality and the GBD									
				Difference (%): (FFQ−DR)/DR*100^†^					Correlation coefficient between the FFQ and DR				
				Number of foods with a difference between the FFQ and DR within 20%	Low		High		Mean	Low		High	
					Food	Value	Food	Value		Food	Value	Food	Value
47-item FFQ^(41)^	20	20	10	2	Milk	7	Seaweed	−92	0.47	Potato	0.17	Milk	0.76
	20	20	10	2	Milk	8	Seaweed	−82	0.44	Seaweed	0.23	Rice	0.61
DHQ^(43)^	33	27	17	11	Grains	0	Potato	−53	0.43	Seaweed	0.08	Dairy	0.72
	33	27	17	10	Meat, total	−3	Sugar and sweeteners	36	0.40	Potato	0.13	Meat, total	0.66
BDHQ^(43)^	29	25	15	10	Seaweed	1	Beans	52	0.45	Potato	0.21	Rice	0.61
	29	25	15	10	Grains	1	Sugar and sweeteners	−39	0.42	Potato Seaweed	0.17	Meat, total	0.63
MDHQ^(49)^	24	10	10	4	Soft drinks	0	Confectionaries	104	0.43	Meat	0.21	Eggs	0.66
	24	10	10	8	Soft drinks	0	Confectionaries	136	0.45	Potato	0.21	Dairy products	0.61
FFQ in JACC^(52)^	40	40	10	3	Egg	−8	Meat, beef	375	0.42	Rice	0.16	Milk	0.72
	40	40	10	4	Egg	1	Meat, beef	238	0.41	Seaweed	0.00	Meat, pork	0.75
JPHC FFQ at baseline^(55)^	17	16	13	1	Grains	10	Dairy	104	0.29	Seasonings and spices	0.08	Fruit	0.55
	17	16	13	3	Grains	−2	Dairy	95	0.28	Seasonings and spices	0.08	Dairy	0.46
JPHC_5y^(57)^	19	18	15	7	Seeds and nuts	−1	Seasonings and spices	−88	0.34	Seaweed	0.08	Beans	0.53
	19	18	15	6	Beans	0	Seasonings and spices	−85	0.31	Seaweed	0.06	Dairy	0.64
FFQ in JPHC-NEXT^(65)^	29	23	18	7	Fats and oils	1	Sugar and sweeteners	−91	0.45	Seaweed Seasonings and spices	0.25	Fruit	0.75
	29	23	18	10	Seaweed	0	Dairy	114	0.40	Fats and oils	0.06	Beans	0.61
Short-FFQ in JPHC-NEXT^(65)^	25	20	15	7	Fruit	2	Seasonings and spices	−88	0.44	Fats and oils	0.16	Beans	0.63
	25	20	15	7	Fruit	7	Seasonings and spices	−87	0.42	Seasonings and spices	0.16	Dairy	0.73
Short version FFQ^(68)^	17	17	17	8	Mushroom	1	Confectionaries	88	0.31	Seeds and nuts	0.13	Mushrooms	0.80
Maruyama FFQ^(69)^	11	11	11	3	Fruit	3	Beans	83	0.52	Meat, total	0.24	Grains	0.75

FFQ, Food frequency questionnaire; DR, Dietary record; GBD, Global burden of disease; NHNS, National Health and Nutrition Survey; 47-item FFQ, 47-item short food frequency questionnaire; DHQ, Self-administered diet history questionnaire; BDHQ, Brief-type self-administered diet history questionnaire; MDHQ, Meal-based Diet History Questionnaire; JPHC FFQ at baseline, 44-item food frequency questionnaire; JPHC_5y, JPHC FFQ at 5-year follow-up; FFQ in JPHC-NEXT, Long-FFQ in the Japan Public Health Centre-based prospective Study for the Next Generation (JPHC-NEXT); Short-FFQ in JPHC-NEXT, 66-item food frequency questionnaire for the Japan Public Health Centre-based prospective Study for the Next Generation (JPHC-NEXT) follow-up survey; Short version FFQ, Short version of the Shizuoka Prefecture version of the Food Intake Frequency Questionnaire; Maruyama FFQ, Maruyama food frequency questionnaire

† Differences were evaluated in absolute values.

The range of the mean CCs of nutrient intake was 0.35 (JPHC FFQ at baseline) to 0.57 (DHQ). The mean proportion of nutrients with a CC of 0.5 or higher, which implies that a tool is reliable for measuring dietary intake using the relevant dietary questionnaire^(35)^, was 37%. Poly unsaturated fatty acids tended to have low CCs. For nutrients., both the DHQ (group-level comparison: approximately 95%, CCs: approximately 70%) and BDHQ (group-level comparison: approximately 90%, CCs: approximately 70%) had high values for both group-level comparison and CCs, whereas the FFQ in JPHC-NEXT, Short-FFQ in JPHC-NEXT, and Maruyama FFQ tended to have high CCs but low group-level comparison results.

The number of food groups reported as a main group in the NHNS or as a dietary risk in terms of the global burden of disease and mortality that were validated in each questionnaire ranged from 10 (47-item FFQ, MDHQ, and FFQ in JACC) to 18 (FFQ in JPHC-NEXT). Among these food groups, the number of group-level comparisons within 20% against reference methods ranged from one (JPHC FFQ at baseline) to 11 (DHQ). The mean proportion of food groups with a group-level comparison result within 20% was 43%. The highest number of group-level comparisons exceeding 20% against reference methods was 37 food groups in FFQ in JACC. Additionally, the range of the mean CCs for food group intakes was 0.28 (JPHC FFQ at baseline) to 0.52 (Maruyama FFQ). The mean proportion of food groups with a CC of 0.5 or higher was 31%. The CCs for grains including rice, fruit, and dairy were high (mean CC: 0.51, 0.53, and 0.61, respectively), while the CCs for seaweed, seasonings, and potato were low (mean CC: 0.22, 0.17, and 0.25, respectively) in all 11 questionnaires. For food groups, the BDHQ (group-level comparison: approximately 70%, CCs: approximately 45%) had high values for both group-level comparison and CCs. These trends were similar in the additional articles — the validity results reported in these articles are shown in Supplementary Table 4.

## Discussion

This scoping review sought the existence of questionnaires to assess dietary intakes of the Japanese population, with a view to assess their suitability for the Japanese NHNS. To our knowledge, this is the first scoping review to identify questionnaires, as opposed to identification of article-based, that estimate dietary intake in healthy adults within a country — this allows us to compare questionnaires and identify a suitable tool for the intended purpose given that the validity of a dietary questionnaire can be influenced by the number of validation studies that have been conducted. Among 32 articles that were of good quality, we identified 11 dietary questionnaires. However, validity in terms of nutrient and food group intake varied among the questionnaires, and there was a range of mean intake values (group-level comparison) and CCs that were used as indicators within each questionnaire.

To date, there have been two article-based reviews of FFQs in Japan. One was reported in 2009, in which 21 articles were extracted (including articles reporting on the same FFQ)^(24)^. The other reviewed 50 articles published until 2017^(31)^. The number of articles cannot be compared because the second review included studies that used biomarkers as the reference method^(31)^; nonetheless, fewer studies used a dietary record or 24-h recall as the reference dietary assessment method (n = 15) than in the current review (n = 32). This may be owing to the publication of seven articles after 2017 and the different search formulae used. The present study used a search formula that broadly captured terms related to ‘validity’ and ‘questionnaire’. This enabled us to identify more studies that assessed validity against dietary record and 24-h recall reference methods. The most important difference was that the two previous reviews aimed to review validation studies of FFQs developed in Japan, whereas the current review aims to identify FFQs that can be used for the NHNS in Japan (calculating representative values of nutrient intakes for Japanese aged 1 year and older).

The present review included studies conducted among Japanese adults, the primary target population of the NHNS. Most participants in the validity studies were middle-aged or older, because many of the questionnaires (6 of 11) were developed for use in cohort studies that targeted middle-aged or older individuals, and the validity studies were conducted among a subsample of the main cohort study^(52,55–57,65,69)^. The number of items in the questionnaires ranged from 40 to 196, and for only three questionnaires, the time required for completion was reported. Given that the time required for completion may be one factor indicative of the burden on participants, future reports are anticipated. Additionally, there is no articles which described the necessity of supports by the interviewers that is labor-intensive for investigators (data not shown), thus the future study should assess it.

Use of the dietary record method in most validity studies reflects its common use in Japan, and may be related to it being the tool used in the Japanese NHNS, which is contrary to many other countries that use the 24-h recall in their national nutrition surveys^(70)^. The dietary record and weighed food record are recommended as the first-choice reference method when validating dietary questionnaires^(23)^, indicating that the results of the validity studies extracted in this study are rather reliable.

Group-level comparison was performed and CCs calculated in all main and most additional articles in this review (not reported in additional articles: group-level comparison, n = 4; CCs, n = 1). CCs, which measure the strength and direction of an association between two different measurements at the individual level, are most commonly used as indicators of validity in studies of dietary questionnaires^(35)^. However, as CCs do not measure the degree of agreement between the two methods, the assessment of indicators that reflect agreement between two measurements at the group level (“group-level comparison” and “Bland–Altman plot”) is also important^(35)^. These results may explain the good quality and excellent validation design of the studies on dietary questionnaires extracted in the present review.

The dietary questionnaires extracted in this scoping review were all reported to be valid (especially in terms of ranking ability for nutrients and foods intakes); nevertheless, results varied by nutrient and food group, similar to reports on questionnaires in other countries^(26,71)^. Despite the ease of understanding intake frequency by participants for both fruit and dairy, the inconsistency evident within the dairy group less clear. Fruit and dairy have the potential to indicate higher CCs because the intake frequency is easier to understand^(30)^. On the other hand, as a factor that causes differences in agreement of group level comparison, fruits are easier to set portion sizes and answer (e.g. 1/2 apple)^(31)^, whereas dairy, especially milk which accounts for the majority of Japanese dairy intake^(13)^, may be consumed in small amounts (e.g. used for cooking) or large amounts (e.g. in beverages)^(23)^.

Additionally, for grains, including rice, many articles had correlation coefficients higher than 0.5 and group level comparison within 20% in the present study. Most of the FFQs request more detail on grains items including rice as the staple food of the Japanese population^(72)^. Higher agreement in carbohydrate intake was found between validation studies, which may be related to more details collected through the questionnaire^(23)^. With grains (especially rice) making up 60% of the Japanese dietary intake^(13)^, this reflects the importance of collecting greater details of frequently-consumed items for more accurate outcomes. On the other hand, the validity results for seaweed, seasonings, and potato intake did not show good agreement in group-level comparisons or CCs. These foods require cooking and have multiple uses; thus, they are consumed in all three major dietary patterns of the Japanese population (healthy [characterized by higher intakes of mushrooms, seaweeds, vegetables, pickles potatoes, fruits, and pulses], prudent [characterized by higher intakes of mushrooms, seaweeds, vegetables, potatoes, fruits, and pulses] and Japanese [characterized by higher intakes of mushrooms, seaweeds, potatoes, vegetables, pickles, pulses, seasonings, fruits, and fish and shellfish] patterns)^(73)^, but with low daily intake^(13)^. Additionally, it has been reported that the Japanese diet is characterized by the use of various ingredients, seasonings, and cooking methods, which may influence responses on food intake frequency^(74)^. These factors may complicate the estimation of portion size^(75)^ in FFQs that require recall^(35,76)^, which in turn may affect the estimation of nutrient and food group intakes. Simultaneously, the lower CCs of seasonings and spices compared to those obtained in Western countries (0.6–0.7)^(77)^ and Iran (0.39)^(30)^ may be a result of the higher complexity of the Japanese diet found to contain more ingredients than Western or Iranian diets^(74)^.

Four FFQs (FFQ in JACC, FFQ in JPHC-NEXT, Short-FFQ in JPHC-NEXT, and Maruyama FFQ) tended to have high CCs and low group-level comparison. This may be due to the fact that the ability to categorize populations (quartiles or quintiles) by nutrient and food group intake was a priority in order to examine the association between dietary intake and outcomes in the development of questionnaires used in cohort studies^(78)^.

Another factor is that the validation of these dietary questionnaires was conducted in a subsample of the main cohort, and the fact that the FFQs were conducted after the dietary records may have affected the CCs^(23)^. If the FFQ is administered after the dietary record method, there is concern that subjects may respond to the FFQ under the influence of their most recent dietary record^(23)^. It is important to carefully scrutinize publications related to questionnaire development and validation studies to determine whether the original purpose of the questionnaire meets the requirements of the new study^(23)^. Given that the NHNS requires the assessment of average dietary intake in the Japanese population, it will be necessary to select the dietary questionnaire that has the high values for group-level comparisons and CCs.

Considering the perspective of the dietary questionnaire used in the NHNS, the intake of salt, vegetables, and fruit — for which the government has set targets (Health Japan 21) because their intake is considered a challenge in the Japanese population^(79)^ — is monitored in the NHNS results. Hence, ensuring accuracy in validity measurement in terms of these intakes is even more important. Considering the validity results for salt, vegetable, and fruit intake, group-level comparisons within 20% and CCs above 0.5 for studies of ‘Good’ quality, or close to but not exactly 0.5 for those of ‘Acceptable’ quality^(35)^, were obtained for the BDHQ and MDHQ. These results may provide one basis for consideration of the use of these tools in the current NHNS.

Moreover, the findings of validation studies (particularly summary values, figures, and numbers) should be carefully interpreted. For example, CCs increase as collection days increase in reference methods, indicating a higher agreement/correlation with a greater amount of data collected^(78,80)^. Further, there is no consensus on assessing and interpreting the Bland–Altman plot in validation studies of dietary assessment questionnaires^(35)^. Bland–Altman analysis reflects the presence, direction, and extent of bias, as well as the level of agreement between two measures^(35)^; however, the decision about what is acceptable agreement is a clinical one; statistics alone cannot answer the question^(81)^. This is partially reflected by the fact that all the dietary assessment questionnaires were considered ‘valid’ in each of the original articles. Thus, the present review should be considered a scientific basis (not an absolute answer) for selecting a suitable dietary assessment questionnaire for future research, including for the NHNS. In the future, it will be necessary to verify whether the 11 questionnaires identified in the present study can be used in the NHNS participants or sub-sample of them (not only in terms of validity and feasibility but also in terms of participation rates, and consistency with previous NHNS results). Additionally, although 11 questionnaires were extracted in the present study, the dietary patterns, energy, macro nutrient and salt intake among Japanese have changed over time^(82–84)^. It will be necessary to periodically examine the feasibility of using the questionnaires to monitor changes in the dietary intake of the Japanese population through the NHNS.

The FFQ has been used in national surveys in several foreign countries^(70)^. For the characteristics of its use, not only the association between dietary intake and risk of NCDs^(85)^ but also pesticide residue exposure status has also been examined^(86)^. To date, pesticide residues have not been evaluated in the NHNS. In Japan, pesticide residues are mainly evaluated using blood and urine^(87,88)^, and there are few studies on dietary exposure for representative Japanese populations^(89,90)^. The evaluation of pesticide residues using blood and urine do not provide estimation of long-term dietary exposure. If dietary questionnaires could be employed as part of the NHNS on a regular basis, the NHNS will provide important information that can be used to assess domestic food safety (e.g. long-term dietary exposure to pesticide residues), as well as basic data for establishing dietary guidelines and updating food composition tables.

The major strength of this review is that we were able to extract dietary questionnaires that can be used for intake assessment in healthy Japanese adults (the primary targets of the NHNS) from articles published in both English and Japanese.

However, this scoping review has several limitations. First, it is unknown whether these same validation results would be obtained when the dietary questionnaire is used in populations other than the population in which it was validated. When using the dietary questionnaire in the NHNS, a pilot study should first be conducted to validate the new questionnaire in the NHNS population. Further, it may be important to evaluate its validity in a (preferably randomly selected) subsample of participants in the same year conducting the NHNS. In terms of calibration capability, accuracy is increased when a dietary questionnaire is used in conjunction with a detailed dietary survey (dietary record or 24-h recall)^(91)^. Therefore, it may be necessary to continue to use the dietary record method, even if only for a subsample rather than for all participants. The use of web-based dietary records or 24-h recalls, which are reported to reduce problems related to cost and participation rates, should be considered^(92,93)^. Second, although the employed strategy in this scoping review was to use wide search terms, search multiple relevant databases, and manually search reference lists, we may not have captured all relevant articles. Third, in order to cover the overall intake of nutrients and food groups, validation studies based on biomarkers, which are often referred to as the gold standard but are only available for a limited number of dietary components, were omitted. It is necessary to select a dietary questionnaire after reviewing studies in which the validity of dietary questionnaires was examined against biomarkers. Fourth, the quality assessment was modified for this review. It should therefore be noted that it cannot be compared with other studies that have assessed quality using the EURECCA tool^(28,30)^. Finally, we did not examine the information about portion size in each questionnaire. However, it has been reported that there is little difference between the absence and presence of a specified portion size^(23)^.

## Conclusions

This scoping review identified 11 dietary questionnaires that can assess the dietary intake of Japanese adults. All the extracted dietary questionnaires were reported to be valid; nonetheless, the group-level comparison and CC values — which are indicators of validity in terms of nutrient and food group intake — varied. When using an existing dietary questionnaire in the NHNS, the results and design of the validation study about the relevant tool should be considered, and a dietary questionnaire with high group-level comparison and CC values on the intended assessment items (especially those items for which NHNS data is used in evaluating target items) should be selected. In the future, the validation of the questionnaire needs to be assessed in NHNS participants.

## Supporting information

Matsumoto et al. supplementary material Matsumoto et al. supplementary material

## Supplementary material

To view supplementary material for this article, please visit https://doi.org/10.1017/jns.2024.1
