# Supplementary material for: A scoping review of dietary assessment questionnaires potentially suitable for assessing habitual dietary intake in the National Health and Nutrition Survey, Japan
Source: J Nutr Sci. 2024 Feb 12;13:e8. doi: 10.1017/jns.2024.1 (PMC10877143; doi:10.1017/jns.2024.1)
Supplement: Matsumoto et al. supplementary material [file S2048679024000016sup001.docx]

| Supplemental Table 1. Search terms for a scoping review of validating dietary questionnaires used in the NHNS, Japan | | | | | | | | | | | | | | |
| --- | --- | --- | --- | --- | --- | --- | --- | --- | --- | --- | --- | --- | --- | --- |
| Concept | Terms which entered into the search formula | | | | |  |  |  |  |  |  |  |  |  |
| "Japanese" | (Japanese OR Japan OR "Japanese population") | | | | |  |  |  |  |  |  |  |  |  |
| “FFQs” or “dietary history questionnaires” | (("food frequency questionnaire*" OR FFQ OR FFQs OR "food frequency method*") OR ("diet history questionnaire*" OR "diet history method*")) | | | | | | | | | | | | |  |
|  |  |  |  |  |  |  |  |  |  |  |  |  |  |  |
| “dietary record” or  “24-hour recall” | (("dietary record" OR "dietary records" OR "diet record" OR "diet records" OR "food diary" OR "food diaries" OR "food record" OR "food records") OR (recall OR recalls)) | | | | | | | | | | | | |  |
|  |  |  |  |  |  |  |  |  |  |  |  |  |  |  |
| “validation” | (validity OR validation OR validate OR validated OR reliability OR reliable OR comparison OR compare OR compared OR accura*) | | | | | | | | | | | | |  |
|  |  |  |  |  |  |  |  |  |  |  |  |  |  |  |

Supplementary Table 2. The results of the validity assessment of dietary questionnaires to estimate nutrient intake among Japanese adults in main articles

| FFQ | Participant characteristics | | | Energy-adjustment methods in mean value calculation 1: Crude 2: Density method 3: Residual method | Statistics: Correlations  1: Correlation 2: Adjusted correlation (energy) 3: Deattenuated or intraclass correlation | Type of correlation coefficient P: Pearson S: Spearman | Energy (kcal/d) | | | | |
| --- | --- | --- | --- | --- | --- | --- | --- | --- | --- | --- | --- |
|  |  |  |  |  |  |  | Group level (mean or median) | | | | Correlation coefficient |
|  | Sex M: Male F: Female | Age (years) | n |  |  |  | Mean [median] (FFQ) | Mean [median] (DR) | Difference (%): FFQ−DR)/DR*100 | Statistics: 1: No difference 2: Difference 3: Not tested |  |
| 47-item FFQ ^(38)^ | M | 30-68 | 73 | 1 | 3 | P | 1987 | 2342 | -15 | 2 | 0.49 |
|  | F | 30-68 | 129 | 1 | 3 | P | 1639 | 1924 | -15 | 2 | 0.44 |
| 47-item FFQ ^(41)^ | M | 35-69 | 143 | 3 | - | - | 2295 | 2253 | 2 | 3 | - |
|  | F | 35-69 | 145 | 3 | - | - | 1942 | 1752 | 11 | 3 | - |
| DHQ ^(44)^ | M | 32-76 | 92 | 3 | 3 | P | 2227 | 2343 | -5 | 2 | 0.42 |
|  | F | 31-69 | 92 | 3 | 3 | P | 1878 | 1846 | 2 | 1 | 0.32 |
| BDHQ ^(44)^ | M | 32-76 | 92 | 3 | 3 | P | 2133 | 2343 | -9 | 2 | 0.24 |
|  | F | 31-69 | 92 | 3 | 3 | P | 1713 | 1846 | -7 | 2 | 0.31 |
| MDHQ ^(51)^ | M | 30-69 | 111 | 3 | 2 | P | 1912 | 2223 | -14 | 2 | 0.39 |
|  | F | 30-69 | 111 | 3 | 2 | P | 1434 | 1769 | -19 | 2 | 0.38 |
| FFQ in JACC ^(52)^ | M | 45–77 | 55 | 1 | 3 | S | 2009 | 2386 | -16 | 3 | 0.55 |
|  | F | 47–76 | 58 | 1 | 3 | S | 1359 | 1857 | -27 | 3 | 0.36 |
| JPHC FFQ at baseline ^(55)^ | M | 40-59 | 94 | 3 | 2 | S | 1993 | 2334 | -15 | 3 | 0.52 |
|  | F | 40-59 | 107 | 3 | 2 | S | 1355 | 1814 | -25 | 3 | 0.38 |
| JPHC_5y ^(56)^ | M | - | 102 | 3 | 2 | S | 2352 | 2347 | 0 | 3 | 0.55 |
|  | F | - | 113 | 3 | 2 | S | 2018 | 1820 | 11 | 3 | 0.44 |
| FFQ in JPHC-NEXT ^(65)^ | M | 40-74 | 98 | 1 | 3 | S | 2390 | 2315 | 3 | 1 | 0.45 |
|  | F | 40-74 | 142 | 1 | 3 | S | 2036 | 1805 | 13 | 2 | 0.17 |
| Short-FFQ in JPHC-NEXT ^(65)^ | M | 40-74 | 92 | 1 | 3 | S | 1857 | 2345 | -21 | 2 | 0.49 |
|  | F | 40-74 | 136 | 1 | 3 | S | 1382 | 1810 | -24 | 2 | 0.16 |
| Short version FFQ ^(68)^ | MF | mean: 55.7 | 491 | 3 | 2 | P | 1874 | 1756 | 7 | 3 | 0.36 |
| Maruyama FFQ ^(69)^ | MF | 47-78 | 58 | 3 | 2 | S | 1907 | 2043 | -7 | 3 | 0.58 |

| FFQ | Protein (g/d) | | | | | Animal protein (g/d) | | | | |
| --- | --- | --- | --- | --- | --- | --- | --- | --- | --- | --- |
|  | Group level (mean or median) | | | | Correlation coefficient | Group level (mean or median) | | | | Correlation coefficient |
|  | Mean [median] (FFQ) | Mean [median] (DR) | Difference (%): FFQ−DR)/DR*100 | Statistics: 1: No difference 2: Difference 3: Not tested |  | Mean [median] (FFQ) | Mean [median] (DR) | Difference (%): FFQ−DR)/DR*100 | Statistics: 1: No difference 2: Difference 3: Not tested |  |
| 47-item FFQ ^(38)^ | 61 | 88 | -31 | 2 | 0.50 | - | - | - | - | - |
|  | 55 | 75 | -26 | 2 | 0.36 | - | - | - | - | - |
| 47-item FFQ ^(41)^ | - | - | - | - | - | - | - | - | - | - |
|  | - | - | - | - | - | - | - | - | - | - |
| DHQ ^(43)^ | 72 | 83 | -14 | 2 | 0.31 | - | - | - | - | - |
|  | 66 | 70 | -5 | 2 | 0.52 | - | - | - | - | - |
| BDHQ ^(44)^ | 75 | 83 | -10 | 2 | 0.41 | - | - | - | - | - |
|  | 67 | 70 | -3 | 2 | 0.38 | - | - | - | - | - |
| MDHQ ^(51)^ | 57.9 | 79.0 | -27 | 2 | 0.31 | - | - | - | - | - |
|  | 48.9 | 64.0 | -24 | 2 | 0.48 | - | - | - | - | - |
| FFQ in JACC ^(52)^ | 65 | 95 | -32 | 3 | 0.25 | - | - | - | - | - |
|  | 55 | 80 | -31 | 3 | 0.49 | - | - | - | - | - |
| JPHC FFQ at baseline ^(55)^ | 65 | 93 | -30 | 3 | 0.28 | - | - | - | - | - |
|  | 52 | 76 | -31 | 3 | 0.34 | - | - | - | - | - |
| JPHC_5y ^(56)^ | 90 | 93 | -4 | 3 | 0.30 | - | - | - | - | - |
|  | 83 | 76 | 9 | 3 | 0.27 | - | - | - | - | - |
| FFQ in JPHC-NEXT ^(65)^ | 79 | 84 | -6 | 1 | 0.40 | - | - | - | - | - |
|  | 77 | 70 | 10 | 2 | 0.33 | - | - | - | - | - |
| Short-FFQ in JPHC-NEXT ^(65)^ | 61 | 85 | -28 | 2 | 0.35 | - | - | - | - | - |
|  | 53 | 70 | -24 | 2 | 0.43 | - | - | - | - | - |
| Short version FFQ ^(68)^ | 65 | 64 | 1 | 3 | 0.33 | - | - | - | - | - |
| Maruyama FFQ ^(69)^ | 77 | 79 | -3 | 3 | 0.48 | 41.1 | 41.6 | -1 | 3 | 0.41 |

| FFQ | Fat (g/d) | | | | | Animal fat (g/d) | | | | |
| --- | --- | --- | --- | --- | --- | --- | --- | --- | --- | --- |
|  | Group level (mean or median) | | | | Correlation coefficient | Group level (mean or median) | | | | Correlation coefficient |
|  | Mean [median] (FFQ) | Mean [median] (DR) | Difference (%): FFQ−DR)/DR*100 | Statistics: 1: No difference 2: Difference 3: Not tested |  | Mean [median] (FFQ) | Mean [median] (DR) | Difference (%): FFQ−DR)/DR*100 | Statistics: 1: No difference 2: Difference 3: Not tested |  |
| 47-item FFQ ^(38)^ | 47.1 | 66.1 | -29 | 2 | 0.62 | - | - | - | - | - |
|  | 48.4 | 59.2 | -18 | 2 | 0.48 | - | - | - | - | - |
| 47-item FFQ ^(41)^ | - | - | - | - | - | - | - | - | - | - |
|  | - | - | - | - | - | - | - | - | - | - |
| DHQ ^(43)^ | 57.4 | 64.1 | -10 | 2 | 0.57 | - | - | - | - | - |
|  | 57.3 | 56.1 | 2 | 1 | 0.62 | - | - | - | - | - |
| BDHQ ^(44)^ | 51.7 | 64.1 | -19 | 2 | 0.64 | - | - | - | - | - |
|  | 48.0 | 56.1 | -14 | 2 | 0.61 | - | - | - | - | - |
| MDHQ ^(51)^ | 53.1 | 72.9 | -27 | 2 | 0.30 | - | - | - | - | - |
|  | 48.5 | 60.2 | -19 | 2 | 0.43 | - | - | - | - | - |
| FFQ in JACC ^(52)^ | 35.5 | 56.3 | -37 | 3 | 0.37 | - | - | - | - | - |
|  | 33.4 | 52.0 | -36 | 3 | 0.50 | - | - | - | - | - |
| JPHC FFQ at baseline ^(55)^ | 34.7 | 59.2 | -41 | 3 | 0.30 | - | - | - | - | - |
|  | 31.8 | 52.7 | -40 | 3 | 0.41 | - | - | - | - | - |
| JPHC_5y ^(56)^ | 66.1 | 59.2 | 12 | 3 | 0.52 | - | - | - | - | - |
|  | 64.5 | 52.9 | 22 | 3 | 0.46 | - | - | - | - | - |
| FFQ in JPHC-NEXT ^(65)^ | 61.1 | 62.6 | -2 | 1 | 0.53 | - | - | - | - | - |
|  | 64.5 | 54.6 | 18 | 2 | 0.33 | - | - | - | - | - |
| Short-FFQ in JPHC-NEXT ^(65)^ | 42.3 | 63.9 | -34 | 2 | 0.50 | - | - | - | - | - |
|  | 38.0 | 54.7 | -31 | 2 | 0.29 | - | - | - | - | - |
| Short version FFQ ^(68)^ | 47.1 | 44.0 | 7 | 3 | 0.33 | - | - | - | - | - |
| Maruyama FFQ ^(69)^ | 53.3 | 54.7 | -3 | 3 | 0.57 | 24.5 | 27.7 | -12 | 3 | 0.63 |

| FFQ | Saturated fatty acid (g/d) | | | | | Monounsaturated fatty acid (g/d) | | | | |
| --- | --- | --- | --- | --- | --- | --- | --- | --- | --- | --- |
|  | Group level (mean or median) | | | | Correlation coefficient | Group level (mean or median) | | | | Correlation coefficient |
|  | Mean [median] (FFQ) | Mean [median] (DR) | Difference (%): FFQ−DR)/DR*100 | Statistics: 1: No difference 2: Difference 3: Not tested |  | Mean [median] (FFQ) | Mean [median] (DR) | Difference (%): FFQ−DR)/DR*100 | Statistics: 1: No difference 2: Difference 3: Not tested |  |
| 47-item FFQ ^(38)^ | 11.3 | 16.6 | -32 | 2 | 0.64 | 17.5 | 23.1 | -24 | 2 | 0.43 |
|  | 12.4 | 16.0 | 22 | 2 | 0.42 | 16.9 | 19.8 | -15 | 2 | 0.34 |
| 47-item FFQ ^(41)^ | - | - | - | - | - | - | - | - | - | - |
|  | - | - | - | - | - | - | - | - | - | - |
| DHQ ^(43)^ | 14.8 | 17.4 | -15 | 2 | 0.60 | 20.4 | 22.7 | -10 | 2 | 0.63 |
|  | 15.5 | 15.8 | -2 | 1 | 0.75 | 19.9 | 19.5 | 2 | 1 | 0.57 |
| BDHQ ^(44)^ | 13.4 | 17.4 | -23 | 2 | 0.63 | 18.2 | 22.7 | -20 | 2 | 0.66 |
|  | 13.0 | 15.8 | -18 | 2 | 0.70 | 16.6 | 19.5 | -15 | 2 | 0.66 |
| MDHQ ^(51)^ | 15.7 | 20.9 | -25 | 2 | 0.40 | 19.8 | 28.5 | -31 | 2 | 0.26 |
|  | 14.9 | 18.0 | -17 | 2 | 0.49 | 17.6 | 22.9 | -23 | 2 | 0.33 |
| FFQ in JACC ^(52)^ | - | - | - | - | - | - | - | - | - | - |
|  | - | - | - | - | - | - | - | - | - | - |
| JPHC FFQ at baseline ^(55)^ | 12.8 | 16.0 | -20 | 3 | 0.42 | 10.6 | 19.6 | -46 | 3 | 0.23 |
|  | 12.2 | 14.8 | -17 | 3 | 0.50 | 9.6 | 17.4 | -45 | 3 | 0.39 |
| JPHC_5y ^(56)^ | - | - | - | - | - | - | - | - | - | - |
|  | - | - | - | - | - | - | - | - | - | - |
| FFQ in JPHC-NEXT ^(65)^ | 17.5 | 17.1 | 3 | 1 | 0.48 | 22.6 | 22.8 | -1 | 1 | 0.55 |
|  | 19.0 | 15.2 | 26 | 2 | 0.46 | 23.6 | 19.1 | 23 | 2 | 0.21 |
| Short-FFQ in JPHC-NEXT ^(65)^ | 12.1 | 17.4 | -30 | 2 | 0.47 | 15.4 | 23.3 | -34 | 2 | 0.45 |
|  | 10.6 | 15.2 | -30 | 2 | 0.44 | 14.0 | 19.2 | -27 | 2 | 0.21 |
| Short version FFQ ^(68)^ | 13.4 | 11.3 | 19 | 3 | 0.38 | 16.4 | 14.4 | 14 | 3 | 0.27 |
| Maruyama FFQ ^(69)^ | 15.2 | 15.7 | -3 | 3 | 0.74 | 17.2 | 18.2 | -5 | 3 | 0.47 |

| FFQ | n -6 polyunsaturated fatty acid (g/d) | | | | | n -3 polyunsaturated fatty acid (g/d) | | | | |
| --- | --- | --- | --- | --- | --- | --- | --- | --- | --- | --- |
|  | Group level (mean or median) | | | | Correlation coefficient | Group level (mean or median) | | | | Correlation coefficient |
|  | Mean [median] (FFQ) | Mean [median] (DR) | Difference (%): FFQ−DR)/DR*100 | Statistics: 1: No difference 2: Difference 3: Not tested |  | Mean [median] (FFQ) | Mean [median] (DR) | Difference (%): FFQ−DR)/DR*100 | Statistics: 1: No difference 2: Difference 3: Not tested |  |
| 47-item FFQ ^(38)^ | 11.8 | 12.8 | -8 | 2 | 0.12 | 2.3 | 3.3 | -30 | 2 | 0.55 |
|  | 11.5 | 11.0 | 4 | 1 | 0.31 | 2.2 | 2.8 | -20 | 2 | 0.23 |
| 47-item FFQ ^(41)^ | - | - | - | - | - | - | - | - | - | - |
|  | - | - | - | - | - | - | - | - | - | - |
| DHQ ^(43)^ | 11.2 | 11.8 | -5 | 2 | 0.54 | 2.8 | 3.0 | -7 | 2 | 0.42 |
|  | 10.8 | 10.3 | 5 | 2 | 0.50 | 2.6 | 2.5 | 8 | 2 | 0.36 |
| BDHQ ^(44)^ | 10.1 | 11.8 | -14 | 2 | 0.63 | 2.8 | 3.0 | -6 | 1 | 0.25 |
|  | 9.0 | 10.3 | -13 | 2 | 0.59 | 2.5 | 2.5 | 0 | 1 | 0.34 |
| MDHQ ^(51)^ | 9.8 | 12.2 | -20 | 2 | 0.22 | 1.98 | 2.71 | -27 | 2 | 0.18 |
|  | 9.0 | 10.1 | -11 | 2 | 0.27 | 1.74 | 2.13 | -18 | 2 | 0.34 |
| FFQ in JACC ^(52)^ | - | - | - | - | - | - | - | - | - | - |
|  | - | - | - | - | - | - | - | - | - | - |
| JPHC FFQ at baseline ^(55)^ | 5.3 | 12.3 | -56 | 3 | 0.14 | 1.3 | 3.5 | -63 | 3 | 0.20 |
|  | 4.6 | 10.8 | -57 | 3 | 0.15 | 1.2 | 3.0 | -60 | 3 | 0.35 |
| JPHC_5y ^(56)^ | - | - | - | - | - | - | - | - | - | - |
|  | - | - | - | - | - | - | - | - | - | - |
| FFQ in JPHC-NEXT ^(65)^ | 11.0 | 10.5 | 5 | 1 | 0.44 | 2.6 | 2.9 | -11 | 2 | 0.38 |
|  | 11.3 | 9.2 | 23 | 2 | 0.28 | 2.7 | 2.4 | 16 | 2 | 0.40 |
| Short-FFQ in JPHC-NEXT ^(65)^ | 7.4 | 10.7 | -31 | 2 | 0.44 | 2.0 | 3.0 | -33 | 2 | 0.36 |
|  | 6.7 | 9.2 | -28 | 2 | 0.24 | 1.9 | 2.4 | -20 | 2 | 0.30 |
| Short version FFQ ^(68)^ | - | - | - | - | - | - | - | - | - | - |
| Maruyama FFQ ^(69)^ | 10.9 | 10.4 | 5 | 3 | 0.35 | 2.6 | 2.5 | 4 | 3 | 0.15 |

| FFQ | Cholesterol (mg/d) | | | | | Carbohydrate (g/d) | | | | |
| --- | --- | --- | --- | --- | --- | --- | --- | --- | --- | --- |
|  | Group level (mean or median) | | | | Correlation coefficient | Group level (mean or median) | | | | Correlation coefficient |
|  | Mean [median] (FFQ) | Mean [median] (DR) | Difference (%): FFQ−DR)/DR*100 | Statistics: 1: No difference 2: Difference 3: Not tested |  | Mean [median] (FFQ) | Mean [median] (DR) | Difference (%): FFQ−DR)/DR*100 | Statistics: 1: No difference 2: Difference 3: Not tested |  |
| 47-item FFQ ^(38)^ | 274 | 424 | -35 | 2 | 0.13 | 293 | 313 | -6 | 2 | 0.86 |
|  | 264 | 345 | -24 | 2 | 0.19 | 227 | 265 | -14 | 1 | 0.64 |
| 47-item FFQ ^(41)^ | - | - | - | - | - | - | - | - | - | - |
|  | - | - | - | - | - | - | - | - | - | - |
| DHQ ^(43)^ | 336 | 392 | -14 | 2 | 0.51 | 303 | 314 | -4 | 2 | 0.70 |
|  | 303 | 334 | -9 | 2 | 0.39 | 260 | 254 | 2 | 2 | 0.62 |
| BDHQ ^(44)^ | 392 | 392 | 0 | 1 | 0.39 | 291 | 314 | -7 | 2 | 0.68 |
|  | 364 | 334 | 9 | 2 | 0.34 | 240 | 254 | -6 | 2 | 0.51 |
| MDHQ ^(51)^ | 257 | 378 | -32 | 2 | 0.46 | 252.9 | 282.1 | -10 | 2 | 0.66 |
|  | 228 | 302 | -25 | 2 | 0.43 | 193.1 | 217.2 | -11 | 2 | 0.67 |
| FFQ in JACC ^(52)^ | - | - | - | - | - | 311 | 330 | -6 | 3 | 0.57 |
|  | - | - | - | - | - | 208 | 268 | -22 | 3 | 0.43 |
| JPHC FFQ at baseline ^(55)^ | 281 | 419 | -33 | 3 | 0.36 | 308 | 314 | -2 | 3 | 0.51 |
|  | 254 | 359 | -29 | 3 | 0.30 | 211 | 256 | -18 | 3 | 0.33 |
| JPHC_5y ^(56)^ | 334 | 418 | -20 | 3 | 0.33 | 305 | 317 | -4 | 3 | 0.56 |
|  | 316 | 356 | -11 | 3 | 0.35 | 275 | 257 | 7 | 3 | 0.37 |
| FFQ in JPHC-NEXT ^(65)^ | 341 | 369 | -8 | 1 | 0.53 | 310 | 300 | 3 | 2 | 0.74 |
|  | 317 | 304 | 4 | 1 | 0.38 | 277 | 248 | 11 | 2 | 0.40 |
| Short-FFQ in JPHC-NEXT ^(65)^ | 294 | 374 | -21 | 2 | 0.45 | 236 | 302 | -22 | 2 | 0.68 |
|  | 215 | 304 | -29 | 2 | 0.46 | 194 | 249 | -22 | 2 | 0.44 |
| Short version FFQ ^(68)^ | 260 | 233 | 12 | 3 | 0.14 | 269 | 251 | 7 | 3 | 0.41 |
| Maruyama FFQ ^(69)^ | 298 | 331 | -10 | 3 | 0.60 | 253 | 287 | -12 | 3 | 0.76 |

| FFQ | Dietary fiber, total (g/d) | | | | | Dietary fiber, soluble (g/d) | | | | |
| --- | --- | --- | --- | --- | --- | --- | --- | --- | --- | --- |
|  | Group level (mean or median) | | | | Correlation coefficient | Group level (mean or median) | | | | Correlation coefficient |
|  | Mean [median] (FFQ) | Mean [median] (DR) | Difference (%): FFQ−DR)/DR*100 | Statistics: 1: No difference 2: Difference 3: Not tested |  | Mean [median] (FFQ) | Mean [median] (DR) | Difference (%): FFQ−DR)/DR*100 | Statistics: 1: No difference 2: Difference 3: Not tested |  |
| 47-item FFQ ^(38)^ | 11.4 | 16.6 | -31 | 2 | 0.36 | 2.1 | 3.7 | -43 | 2 | 0.25 |
|  | 12.4 | 16.6 | -25 | 2 | 0.47 | 2.3 | 2.4 | -39 | 2 | 0.37 |
| 47-item FFQ ^(41)^ | - | - | - | - | - | - | - | - | - | - |
|  | - | - | - | - | - | - | - | - | - | - |
| DHQ ^(43)^ | 12.7 | 15.2 | -16 | 2 | 0.73 | 3.0 | 3.3 | -10 | 2 | 0.67 |
|  | 13.8 | 14.5 | -5 | 2 | 0.71 | 3.3 | 3.2 | 3 | 1 | 0.69 |
| BDHQ ^(44)^ | 13.9 | 15.2 | -9 | 2 | 0.72 | 3.4 | 3.3 | 4 | 1 | 0.65 |
|  | 13.5 | 14.5 | -7 | 2 | 0.68 | 3.5 | 3.2 | 8 | 2 | 0.67 |
| MDHQ ^(51)^ | 10.7 | 13.4 | -20 | 2 | 0.57 | 2.34 | 3.24 | -28 | 2 | 0.45 |
|  | 9.8 | 12.3 | -20 | 2 | 0.52 | 2.16 | 2.94 | -27 | 2 | 0.53 |
| FFQ in JACC ^(52)^ | - | - | - | - | - | - | - | - | - | - |
|  | - | - | - | - | - | - | - | - | - | - |
| JPHC FFQ at baseline ^(55)^ | - | - | - | - | - | 1.2 | 2.1 | -43 | 3 | 0.35 |
|  | - | - | - | - | - | 1.3 | 2.1 | -37 | 3 | 0.42 |
| JPHC_5y ^(56)^ | - | - | - | - | - | - | - | - | - | - |
|  | - | - | - | - | - | - | - | - | - | - |
| FFQ in JPHC-NEXT ^(65)^ | 14.5 | 16.8 | -14 | 2 | 0.66 | 3.4 | 3.7 | -9 | 2 | 0.58 |
|  | 17.6 | 16.5 | 7 | 1 | 0.61 | 4.2 | 3.7 | 12 | 2 | 0.60 |
| Short-FFQ in JPHC-NEXT ^(65)^ | 9.0 | 17.0 | -47 | 2 | 0.65 | 1.9 | 3.8 | -49 | 2 | 0.62 |
|  | 10.7 | 16.6 | -36 | 2 | 0.57 | 2.4 | 3.8 | -35 | 2 | 0.54 |
| Short version FFQ ^(68)^ | 12.1 | 13.2 | -8 | 3 | 0.46 | 2.4 | 2.8 | -14 | 3 | 0.35 |
| Maruyama FFQ ^(69)^ | 12.5 | 16.9 | -26 | 3 | 0.46 | 2.8 | 3.8 | -26 | 3 | 0.20 |

| FFQ | Dietary fiber, insoluble (g/d) | | | | | Vitamin A  (Retinol activity equivalents) (µgRAE/d) | | | | |
| --- | --- | --- | --- | --- | --- | --- | --- | --- | --- | --- |
|  | Group level (mean or median) | | | | Correlation coefficient | Group level (mean or median) | | | | Correlation coefficient |
|  | Mean [median] (FFQ) | Mean [median] (DR) | Difference (%): FFQ−DR)/DR*100 | Statistics: 1: No difference 2: Difference 3: Not tested |  | Mean [median] (FFQ) | Mean [median] (DR) | Difference (%): FFQ−DR)/DR*100 | Statistics: 1: No difference 2: Difference 3: Not tested |  |
| 47-item FFQ ^(38)^ | 8.0 | 12.1 | -34 | 2 | 0.33 | 1052 | 989 | 6 | 1 | 0.27 |
|  | 9.0 | 12.0 | -25 | 2 | 0.46 | 1052 | 1067 | -1 | 1 | 0.22 |
| 47-item FFQ ^(41)^ | - | - | - | - | - | - | - | - | - | - |
|  | - | - | - | - | - | - | - | - | - | - |
| DHQ ^(43)^ | 9.3 | 11.1 | -16 | 2 | 0.73 | 485 | 620 | -22 | 2 | 0.23 |
|  | 10.0 | 10.5 | -5 | 2 | 0.71 | 563 | 572 | -2 | 1 | 0.59 |
| BDHQ ^(44)^ | 10.1 | 11.1 | -9 | 2 | 0.71 | 718 | 620 | 16 | 2 | 0.30 |
|  | 9.7 | 10.5 | -8 | 2 | 0.68 | 713 | 572 | 25 | 2 | 0.57 |
| MDHQ ^(51)^ | 7.72 | 9.73 | -21 | 2 | 0.57 | 354 | 479 | -26 | 2 | 0.26 |
|  | 6.98 | 8.93 | -22 | 2 | 0.48 | 353 | 409 | -14 | 1 | 0.28 |
| FFQ in JACC ^(52)^ | - | - | - | - | - | - | - | - | - | - |
|  | - | - | - | - | - | - | - | - | - | - |
| JPHC FFQ at baseline ^(55)^ | 7.1 | 10.5 | -32 | 3 | 0.43 | - | - | - | - | - |
|  | 6.6 | 9.9 | -33 | 3 | 0.43 | - | - | - | - | - |
| JPHC_5y ^(56)^ | - | - | - | - | - | - | - | - | - | - |
|  | - | - | - | - | - | - | - | - | - | - |
| FFQ in JPHC-NEXT ^(65)^ | 10.6 | 12.4 | -14 | 2 | 0.68 | 642 | 639 | 1 | 1 | 0.35 |
|  | 12.9 | 12.1 | 6 | 1 | 0.60 | 800 | 575 | 39 | 2 | 0.41 |
| Short-FFQ in JPHC-NEXT ^(65)^ | 6.7 | 12.6 | -46 | 2 | 0.64 | 691 | 653 | 6 | 1 | 0.38 |
|  | 7.9 | 12.2 | -35 | 2 | 0.59 | 655 | 583 | 12 | 1 | 0.34 |
| Short version FFQ ^(68)^ | 8.8 | 9.8 | -10 | 3 | 0.47 | 406 | 415 | -2 | 3 | 0.35 |
| Maruyama FFQ ^(69)^ | 9.5 | 12.3 | -23 | 3 | 0.49 | 643 | 689 | -7 | 3 | 0.55 |

| FFQ | Vitamin D (µg/d) | | | | | Vitamin E (mg/d) | | | | |
| --- | --- | --- | --- | --- | --- | --- | --- | --- | --- | --- |
|  | Group level (mean or median) | | | | Correlation coefficient | Group level (mean or median) | | | | Correlation coefficient |
|  | Mean [median] (FFQ) | Mean [median] (DR) | Difference (%): FFQ−DR)/DR*100 | Statistics: 1: No difference 2: Difference 3: Not tested |  | Mean [median] (FFQ) | Mean [median] (DR) | Difference (%): FFQ−DR)/DR*100 | Statistics: 1: No difference 2: Difference 3: Not tested |  |
| 47-item FFQ ^(38)^ | 7.4 | 9.4 | -21 | 2 | 0.65 | 8.6 | 10.1 | -15 | 2 | 0.31 |
|  | 7.2 | 8.0 | -9 | 1 | 0.40 | 8.0 | 9.4 | -8 | 2 | 0.17 |
| 47-item FFQ ^(41)^ | - | - | - | - | - | - | - | - | - | - |
|  | - | - | - | - | - | - | - | - | - | - |
| DHQ ^(43)^ | - | - | - | - | - | 7.7 | 8.2 | -7 | 2 | 0.49 |
|  | - | - | - | - | - | 7.7 | 7.4 | 4 | 1 | 0.47 |
| BDHQ ^(44)^ | - | - | - | - | - | 7.7 | 8.2 | -7 | 2 | 0.54 |
|  | - | - | - | - | - | 7.3 | 7.4 | -1 | 1 | 0.48 |
| MDHQ ^(51)^ | 5.29 | 7.04 | -24.9 | 2 | 0.20 | 6.20 | 8.22 | -25 | 2 | 0.36 |
|  | 4.23 | 5.91 | -28.4 | 2 | 0.51 | 5.73 | 6.89 | -17 | 2 | 0.34 |
| FFQ in JACC ^(52)^ | - | - | - | - | - | - | - | - | - | - |
|  | - | - | - | - | - | - | - | - | - | - |
| JPHC FFQ at baseline ^(55)^ | - | - | - | - | - | - | - | - | - | - |
|  | - | - | - | - | - | - | - | - | - | - |
| JPHC_5y ^(56)^ | - | - | - | - | - | - | - | - | - | - |
|  | - | - | - | - | - | - | - | - | - | - |
| FFQ in JPHC-NEXT ^(65)^ | 9.1 | 11.3 | -20 | 2 | 0.32 | 8.0 | 8.5 | -5 | 1 | 0.52 |
|  | 9.6 | 9.0 | 7 | 1 | 0.49 | 9.3 | 8.0 | 16 | 2 | 0.50 |
| Short-FFQ in JPHC-NEXT ^(65)^ | 8.1 | 11.5 | -29 | 2 | 0.44 | 4.8 | 8.7 | -44 | 2 | 0.41 |
|  | 8.1 | 9.1 | -10 | 1 | 0.47 | 5.6 | 8.1 | -32 | 2 | 0.49 |
| Short version FFQ ^(68)^ | 6.3 | 4.2 | 50 | 3 | 0.33 | 5.6 | 5.6 | 0 | 3 | 0.27 |
| Maruyama FFQ ^(69)^ | 11.5 | 8.6 | 34 | 3 | 0.23 | 6.9 | 8.2 | -15 | 3 | 0.25 |

| FFQ | Vitamin K (µg/d) | | | | | Vitamin B1 (mg/d) | | | | |
| --- | --- | --- | --- | --- | --- | --- | --- | --- | --- | --- |
|  | Group level (mean or median) | | | | Correlation coefficient | Group level (mean or median) | | | | Correlation coefficient |
|  | Mean [median] (FFQ) | Mean [median] (DR) | Difference (%): FFQ−DR)/DR*100 | Statistics: 1: No difference 2: Difference 3: Not tested |  | Mean [median] (FFQ) | Mean [median] (DR) | Difference (%): FFQ−DR)/DR*100 | Statistics: 1: No difference 2: Difference 3: Not tested |  |
| 47-item FFQ ^(38)^ | - | - | - | - | - | 0.69 | 1.18 | -42 | 2 | 0.26 |
|  | - | - | - | - | - | 0.70 | 1.04 | -35 | 2 | 0.10 |
| 47-item FFQ ^(41)^ | - | - | - | - | - | - | - | - | - | - |
|  | - | - | - | - | - | - | - | - | - | - |
| DHQ ^(43)^ | 233 | 236 | -1 | 1 | 0.50 | 0.89 | 1.07 | -17 | 2 | 0.41 |
|  | 259 | 228 | 14 | 2 | 0.50 | 0.84 | 0.90 | -7 | 2 | 0.46 |
| BDHQ ^(44)^ | 302 | 236 | 28 | 2 | 0.59 | 0.87 | 1.07 | -19 | 2 | 0.37 |
|  | 301 | 228 | 32 | 2 | 0.64 | 0.81 | 0.90 | -10 | 2 | 0.45 |
| MDHQ ^(51)^ | 155.7 | 208.6 | -25 | 2 | 0.39 | 0.81 | 1.05 | -23 | 2 | 0.37 |
|  | 162.5 | 195.2 | -17 | 2 | 0.54 | 0.70 | 0.86 | -19 | 2 | 0.28 |
| FFQ in JACC ^(52)^ | - | - | - | - | - | 0.80 | 1.15 | -30 | 3 | 0.33 |
|  | - | - | - | - | - | 0.75 | 1.01 | -26 | 3 | 0.31 |
| JPHC FFQ at baseline ^(55)^ | - | - | - | - | - | 0.91 | 1.30 | -30 | 3 | 0.36 |
|  | - | - | - | - | - | 0.76 | 1.11 | -32 | 3 | 0.22 |
| JPHC_5y ^(56)^ | - | - | - | - | - | 1.27 | 1.32 | -4 | 3 | 0.40 |
|  | - | - | - | - | - | 1.24 | 1.12 | 10 | 3 | 0.41 |
| FFQ in JPHC-NEXT ^(65)^ | 270 | 298 | -9 | 1 | 0.56 | 1.02 | 1.26 | -19 | 2 | 0.35 |
|  | 341 | 294 | 16 | 2 | 0.52 | 1.10 | 1.02 | 8 | 1 | 0.36 |
| Short-FFQ in JPHC-NEXT ^(65)^ | 207 | 303 | -31 | 2 | 0.65 | 1.29 | 0.80 | -38 | 2 | 0.31 |
|  | 251 | 297 | -15 | 2 | 0.53 | 0.76 | 1.03 | -26 | 2 | 0.37 |
| Short version FFQ ^(68)^ | 174 | 191 | -9 | 3 | 0.34 | 0.90 | 0.78 | 15 | 3 | 0.23 |
| Maruyama FFQ ^(69)^ | 227 | 251 | -10 | 3 | 0.54 | - | - | - | - | - |

| FFQ | Vitamin B2 (mg/d) | | | | | Niacin equivalents (mg/d) | | | | |
| --- | --- | --- | --- | --- | --- | --- | --- | --- | --- | --- |
|  | Group level (mean or median) | | | | Correlation coefficient | Group level (mean or median) | | | | Correlation coefficient |
|  | Mean [median] (FFQ) | Mean [median] (DR) | Difference (%): FFQ−DR)/DR*100 | Statistics: 1: No difference 2: Difference 3: Not tested |  | Mean [median] (FFQ) | Mean [median] (DR) | Difference (%): FFQ−DR)/DR*100 | Statistics: 1: No difference 2: Difference 3: Not tested |  |
| 47-item FFQ ^(38)^ | 1.12 | 1.48 | -24 | 2 | 0.57 | - | - | - | - | - |
|  | 1.20 | 1.38 | -11 | 2 | 0.43 | - | - | - | - | - |
| 47-item FFQ ^(41)^ | - | - | - | - | - | - | - | - | - | - |
|  | - | - | - | - | - | - | - | - | - | - |
| DHQ ^(43)^ | 1.36 | 1.48 | -8 | 2 | 0.43 | 18.4 | 21.0 | -12 | 2 | 0.43 |
|  | 1.37 | 1.33 | 3 | 1 | 0.57 | 15.9 | 16.5 | -4 | 1 | 0.57 |
| BDHQ ^(44)^ | 1.43 | 1.48 | -3 | 1 | 0.61 | 19.5 | 21.0 | -7 | 2 | 0.25 |
|  | 1.35 | 1.33 | 2 | 1 | 0.56 | 16.4 | 16.5 | -1 | 1 | 0.40 |
| MDHQ ^(51)^ | 1.03 | 1.31 | -21 | 2 | 0.46 | 16.7 | 20.7 | -19 | 2 | 0.15 |
|  | 0.95 | 1.12 | -15 | 2 | 0.57 | 13.3 | 16.2 | -18 | 2 | 0.35 |
| FFQ in JACC ^(52)^ | 1.23 | 1.64 | -25 | 3 | 0.43 | 12.0 | 19.7 | -39 | 3 | 0.33 |
|  | 1.21 | 1.55 | -22 | 3 | 0.54 | 10.6 | 16.1 | -34 | 3 | 0.47 |
| JPHC FFQ at baseline ^(55)^ | 1.37 | 1.55 | -12 | 3 | 0.43 | 13.7 | 21.8 | -37 | 3 | 0.14 |
|  | 1.29 | 1.37 | -6 | 3 | 0.39 | 10.2 | 16.9 | -40 | 3 | 0.11 |
| JPHC_5y ^(56)^ | 1.78 | 1.55 | 15 | 3 | 0.34 | 21.0 | 21.9 | -4 | 3 | 0.35 |
|  | 1.72 | 1.38 | 25 | 3 | 0.45 | 18.3 | 16.9 | 8 | 3 | 0.15 |
| FFQ in JPHC-NEXT ^(65)^ | 1.46 | 1.68 | -13 | 2 | 0.43 | 24.9 | 23.7 | 5 | 1 | 0.28 |
|  | 1.65 | 1.49 | 10 | 2 | 0.43 | 22.6 | 18.4 | 23 | 2 | 0.32 |
| Short-FFQ in JPHC-NEXT ^(65)^ | 1.14 | 1.71 | -34 | 2 | 0.54 | 20.3 | 24.1 | -16 | 2 | 0.29 |
|  | 1.08 | 1.50 | -28 | 2 | 0.61 | 18.2 | 18.5 | -2 | 1 | 0.18 |
| Short version FFQ ^(68)^ | 1.22 | 1.09 | 12 | 3 | 0.32 | 26.1 | 14.1 | 85 | 3 | 0.33 |
| Maruyama FFQ ^(69)^ | - | - | - | - | - | - | - | - | - | - |

| FFQ | Vitamin B6 (mg/d) | | | | | Vitamin B12 (µg/d) | | | | |
| --- | --- | --- | --- | --- | --- | --- | --- | --- | --- | --- |
|  | Group level (mean or median) | | | | Correlation coefficient | Group level (mean or median) | | | | Correlation coefficient |
|  | Mean [median] (FFQ) | Mean [median] (DR) | Difference (%): FFQ−DR)/DR*100 | Statistics: 1: No difference 2: Difference 3: Not tested |  | Mean [median] (FFQ) | Mean [median] (DR) | Difference (%): FFQ−DR)/DR*100 | Statistics: 1: No difference 2: Difference 3: Not tested |  |
| 47-item FFQ ^(38)^ | - | - | - | - | - | - | - | - | - | - |
|  | - | - | - | - | - | - | - | - | - | - |
| 47-item FFQ ^(41)^ | - | - | - | - | - | - | - | - | - | - |
|  | - | - | - | - | - | - | - | - | - | - |
| DHQ ^(43)^ | 1.3 | 1.5 | -12 | 2 | 0.57 | 8.1 | 9.0 | -11 | 2 | 0.42 |
|  | 1.1 | 1.2 | -6 | 2 | 0.68 | 7.1 | 7.4 | -4 | 1 | 0.58 |
| BDHQ ^(44)^ | 1.5 | 1.5 | 2 | 1 | 0.51 | 9.7 | 9.0 | 8 | 1 | 0.46 |
|  | 1.3 | 1.2 | 8 | 2 | 0.52 | 8.6 | 7.4 | 17 | 2 | 0.39 |
| MDHQ ^(51)^ | 1.05 | 1.38 | -24 | 2 | 0.38 | 4.24 | 5.93 | -29 | 2 | 0.26 |
|  | 0.87 | 1.13 | -23 | 2 | 0.43 | 3.38 | 4.88 | -31 | 2 | 0.45 |
| FFQ in JACC ^(52)^ | - | - | - | - | - | - | - | - | - | - |
|  | - | - | - | - | - | - | - | - | - | - |
| JPHC FFQ at baseline ^(55)^ | - | - | - | - | - | - | - | - | - | - |
|  | - | - | - | - | - | - | - | - | - | - |
| JPHC_5y ^(56)^ | - | - | - | - | - | - | - | - | - | - |
|  | - | - | - | - | - | - | - | - | - | - |
| FFQ in JPHC-NEXT ^(65)^ | 1.6 | 1.8 | -11 | 2 | 0.42 | 7.7 | 9.8 | -21 | 2 | 0.34 |
|  | 1.6 | 1.5 | 11 | 2 | 0.59 | 7.5 | 7.3 | 3 | 1 | 0.35 |
| Short-FFQ in JPHC-NEXT ^(65)^ | 1.3 | 1.9 | -29 | 2 | 0.41 | 7.2 | 9.9 | -28 | 2 | 0.28 |
|  | 1.2 | 1.5 | -19 | 2 | 0.46 | 6.6 | 7.3 | -11 | 2 | 0.46 |
| Short version FFQ ^(68)^ | 1.2 | 1.1 | 10 | 3 | 0.46 | 4.9 | 4.4 | 11 | 3 | 0.29 |
| Maruyama FFQ ^(69)^ | 1.3 | 1.5 | -11 | 3 | 0.43 | 8.8 | 9.9 | -11 | 3 | 0.23 |

| FFQ | Folate (µg/d) | | | | | Pantothenic acid (mg/d) | | | | |
| --- | --- | --- | --- | --- | --- | --- | --- | --- | --- | --- |
|  | Group level (mean or median) | | | | Correlation coefficient | Group level (mean or median) | | | | Correlation coefficient |
|  | Mean [median] (FFQ) | Mean [median] (DR) | Difference (%): FFQ−DR)/DR*100 | Statistics: 1: No difference 2: Difference 3: Not tested |  | Mean [median] (FFQ) | Mean [median] (DR) | Difference (%): FFQ−DR)/DR*100 | Statistics: 1: No difference 2: Difference 3: Not tested |  |
| 47-item FFQ ^(38)^ | 357 | 417 | -14 | 2 | 0.36 | - | - | - | - | - |
|  | 384 | 409 | -6 | 1 | 0.38 | - | - | - | - | - |
| 47-item FFQ ^(41)^ | - | - | - | - | - | - | - | - | - | - |
|  | - | - | - | - | - | - | - | - | - | - |
| DHQ ^(43)^ | 297 | 385 | -23 | 2 | 0.37 | 6.3 | 7.0 | -9 | 2 | 0.61 |
|  | 308 | 349 | -12 | 2 | 0.55 | 6.1 | 6.0 | 2 | 1 | 0.65 |
| BDHQ ^(44)^ | 399 | 385 | 4 | 1 | 0.58 | 7.1 | 7.0 | 1 | 1 | 0.70 |
|  | 378 | 349 | 8 | 2 | 0.62 | 6.5 | 6.0 | 9 | 2 | 0.59 |
| MDHQ ^(51)^ | 237 | 304 | -22 | 2 | 0.47 | 4.81 | 6.20 | -22 | 2 | 0.52 |
|  | 220 | 270 | -19 | 2 | 0.53 | 4.20 | 5.18 | -19 | 2 | 0.63 |
| FFQ in JACC ^(52)^ | - | - | - | - | - | - | - | - | - | - |
|  | - | - | - | - | - | - | - | - | - | - |
| JPHC FFQ at baseline ^(55)^ | - | - | - | - | - | - | - | - | - | - |
|  | - | - | - | - | - | - | - | - | - | - |
| JPHC_5y ^(56)^ | - | - | - | - | - | - | - | - | - | - |
|  | - | - | - | - | - | - | - | - | - | - |
| FFQ in JPHC-NEXT ^(65)^ | 403 | 453 | -11 | 2 | 0.63 | 7.6 | 7.2 | 7 | 1 | 0.62 |
|  | 484 | 445 | 9 | 2 | 0.62 | 8.1 | 6.4 | 27 | 2 | 0.46 |
| Short-FFQ in JPHC-NEXT ^(65)^ | 291 | 461 | -37 | 2 | 0.55 | 5.8 | 7.3 | -20 | 2 | 0.64 |
|  | 313 | 449 | -30 | 2 | 0.55 | 5.3 | 6.4 | -17 | 2 | 0.61 |
| Short version FFQ ^(68)^ | 335 | 309 | 8 | 3 | 0.45 | 5.5 | 5.1 | 7 | 3 | 0.39 |
| Maruyama FFQ ^(69)^ | 312 | 457 | -32 | 3 | 0.58 | - | - | - | - | - |

| FFQ | Vitamin C (mg/d) | | | | | Sodium (mg/d) | | | | |
| --- | --- | --- | --- | --- | --- | --- | --- | --- | --- | --- |
|  | Group level (mean or median) | | | | Correlation coefficient | Group level (mean or median) | | | | Correlation coefficient |
|  | Mean [median] (FFQ) | Mean [median] (DR) | Difference (%): FFQ−DR)/DR*100 | Statistics: 1: No difference 2: Difference 3: Not tested |  | Mean [median] (FFQ) | Mean [median] (DR) | Difference (%): FFQ−DR)/DR*100 | Statistics: 1: No difference 2: Difference 3: Not tested |  |
| 47-item FFQ ^(38)^ | 103 | 123 | -16 | 1 | 0.45 | - | - | - | - | - |
|  | 122 | 136 | -10 | 1 | 0.52 | - | - | - | - | - |
| 47-item FFQ ^(41)^ | - | - | - | - | - | - | - | - | - | - |
|  | - | - | - | - | - | - | - | - | - | - |
| DHQ ^(43)^ | 101 | 110 | -8 | 1 | 0.52 | 4421 | 4902 | -10 | 2 | 0.32 |
|  | 116 | 107 | 8 | 1 | 0.52 | 4215 | 4109 | 3 | 1 | 0.39 |
| BDHQ ^(44)^ | 138 | 110 | 25 | 2 | 0.64 | 4683 | 4902 | -4 | 2 | 0.57 |
|  | 144 | 107 | 35 | 2 | 0.66 | 3912 | 4109 | -5 | 2 | 0.49 |
| MDHQ ^(51)^ | 70 | 90 | -22 | 2 | 0.54 | 3931 | 4335 | -9 | 2 | 0.31 |
|  | 68 | 79 | -14 | 2 | 0.37 | 3221 | 3340 | -4 | 1 | 0.60 |
| FFQ in JACC ^(52)^ | 96 | 123 | -22 | 3 | 0.58 | 2605 | 6328 | -59 | 3 | 0.37 |
|  | 117 | 141 | -17 | 3 | 0.43 | 2482 | 5650 | -56 | 3 | 0.33 |
| JPHC FFQ at baseline ^(55)^ | 86 | 127 | -32 | 3 | 0.38 | 2491 | 5334 | -53 | 3 | 0.33 |
|  | 97 | 135 | -28 | 3 | 0.29 | 2064 | 4662 | -56 | 3 | 0.49 |
| JPHC_5y ^(56)^ | 166 | 129 | 29 | 3 | 0.42 | 5831 | 5334 | 9 | 3 | 0.41 |
|  | 192 | 137 | 40 | 3 | 0.22 | 5437 | 4652 | 17 | 3 | 0.48 |
| FFQ in JPHC-NEXT ^(65)^ | 123 | 142 | -13 | 2 | 0.65 | 4360 | 4570 | -5 | 1 | 0.34 |
|  | 184 | 155 | 18 | 2 | 0.66 | 4480 | 3809 | 18 | 2 | 0.38 |
| Short-FFQ in JPHC-NEXT ^(65)^ | 65 | 145 | -55 | 2 | 0.56 | 2958 | 4622 | -36 | 2 | 0.50 |
|  | 94 | 156 | -40 | 2 | 0.59 | 2876 | 3805 | -24 | 2 | 0.39 |
| Short version FFQ ^(68)^ | 117 | 98 | 19 | 3 | 0.55 | 3671 | 4048 | -9 | 3 | 0.22 |
| Maruyama FFQ ^(69)^ | 95 | 150 | -37 | 3 | 0.59 | 4258 | 3793 | 12 | 3 | 0.48 |

| FFQ | Potassium (mg/d) | | | | | Calcium (mg/d) | | | | |
| --- | --- | --- | --- | --- | --- | --- | --- | --- | --- | --- |
|  | Group level (mean or median) | | | | Correlation coefficient | Group level (mean or median) | | | | Correlation coefficient |
|  | Mean [median] (FFQ) | Mean [median] (DR) | Difference (%): FFQ−DR)/DR*100 | Statistics: 1: No difference 2: Difference 3: Not tested |  | Mean [median] (FFQ) | Mean [median] (DR) | Difference (%): FFQ−DR)/DR*100 | Statistics: 1: No difference 2: Difference 3: Not tested |  |
| 47-item FFQ ^(38)^ | - | - | - | - | - | 508 | 592 | -14 | 2 | 0.49 |
|  | - | - | - | - | - | 566 | 609 | -7 | 1 | 0.59 |
| 47-item FFQ ^(41)^ | - | - | - | - | - | - | - | - | - | - |
|  | - | - | - | - | - | - | - | - | - | - |
| DHQ ^(43)^ | 2403 | 2880 | -17 | 2 | 0.53 | 468 | 573 | -18 | 2 | 0.69 |
|  | 2472 | 2631 | -6 | 2 | 0.53 | 551 | 563 | -2 | 1 | 0.56 |
| BDHQ ^(44)^ | 2900 | 2880 | 1 | 1 | 0.66 | 567 | 573 | -1 | 1 | 0.68 |
|  | 2796 | 2631 | 6 | 2 | 0.59 | 579 | 563 | 3 | 1 | 0.54 |
| MDHQ ^(51)^ | 2146 | 2536 | -15 | 2 | 0.57 | 399 | 505 | -21 | 2 | 0.33 |
|  | 1968 | 2242 | -12 | 2 | 0.60 | 415 | 477 | -13 | 2 | 0.54 |
| FFQ in JACC ^(52)^ | 2052 | 3195 | -36 | 3 | 0.45 | 485 | 682 | -29 | 3 | 0.57 |
|  | 2149 | 3076 | -30 | 3 | 0.45 | 518 | 671 | -23 | 3 | 0.67 |
| JPHC FFQ at baseline ^(55)^ | 2229.5 | 3188.7 | -30 | 3 | 0.38 | 550 | 615 | -10 | 3 | 0.56 |
|  | 2101.8 | 2926.8 | -28 | 3 | 0.37 | 564 | 594 | -5 | 3 | 0.37 |
| JPHC_5y ^(56)^ | 3309 | 3218 | 3 | 3 | 0.39 | 685 | 623 | 10 | 3 | 0.43 |
|  | 3344 | 2949 | 13 | 3 | 0.31 | 699 | 600 | 17 | 3 | 0.47 |
| FFQ in JPHC-NEXT ^(65)^ | 3142 | 3105 | 1 | 1 | 0.48 | 595 | 570 | 4 | 1 | 0.58 |
|  | 3610 | 2968 | 22 | 2 | 0.54 | 754 | 590 | 28 | 2 | 0.42 |
| Short-FFQ in JPHC-NEXT ^(65)^ | 2177 | 3150 | -31 | 2 | 0.46 | 362 | 577 | -37 | 2 | 0.56 |
|  | 2300 | 2992 | -23 | 2 | 0.47 | 365 | 593 | -38 | 2 | 0.60 |
| Short version FFQ ^(68)^ | 2354 | 2191 | 7 | 3 | 0.55 | 451 | 400 | 13 | 3 | 0.45 |
| Maruyama FFQ ^(69)^ | 2454 | 3065 | -20 | 3 | 0.47 | 724 | 664 | 9 | 3 | 0.69 |

| FFQ | Magnesium (mg/d) | | | | | Phosphorus (mg/d) | | | | |
| --- | --- | --- | --- | --- | --- | --- | --- | --- | --- | --- |
|  | Group level (mean or median) | | | | Correlation coefficient | Group level (mean or median) | | | | Correlation coefficient |
|  | Mean [median] (FFQ) | Mean [median] (DR) | Difference (%): FFQ−DR)/DR*100 | Statistics: 1: No difference 2: Difference 3: Not tested |  | Mean [median] (FFQ) | Mean [median] (DR) | Difference (%): FFQ−DR)/DR*100 | Statistics: 1: No difference 2: Difference 3: Not tested |  |
| 47-item FFQ ^(38)^ | - | - | - | - | - | - | - | - | - | - |
|  | - | - | - | - | - | - | - | - | - | - |
| 47-item FFQ ^(41)^ | - | - | - | - | - | - | - | - | - | - |
|  | - | - | - | - | - | - | - | - | - | - |
| DHQ ^(43)^ | 268 | 309 | -13 | 2 | 0.58 | 1079 | 1242 | -13 | 2 | 0.53 |
|  | 255 | 270 | -6 | 2 | 0.64 | 1033 | 1068 | -3 | 2 | 0.51 |
| BDHQ ^(44)^ | 294 | 309 | -5 | 2 | 0.62 | 1159 | 1242 | -7 | 2 | 0.55 |
|  | 264 | 270 | -2 | 1 | 0.59 | 1058 | 1068 | -1 | 1 | 0.41 |
| MDHQ ^(51)^ | 247 | 281 | -12 | 2 | 0.46 | 883 | 1127 | -22 | 2 | 0.35 |
|  | 219 | 244 | -10 | 2 | 0.56 | 774 | 951 | -19 | 2 | 0.52 |
| FFQ in JACC ^(52)^ | - | - | - | - | - | 911 | 1311 | -31 | 3 | 0.52 |
|  | - | - | - | - | - | 817 | 1139 | -28 | 3 | 0.69 |
| JPHC FFQ at baseline ^(55)^ | - | - | - | - | - | 1164 | 1405 | -17 | 3 | 0.56 |
|  | - | - | - | - | - | 955 | 1168 | -18 | 3 | 0.44 |
| JPHC_5y ^(56)^ | - | - | - | - | - | 1423 | 1414 | 1 | 3 | 0.37 |
|  | - | - | - | - | - | 1321 | 1172 | 13 | 3 | 0.42 |
| FFQ in JPHC-NEXT ^(65)^ | 353 | 325 | 9 | 2 | 0.39 | 1247 | 1258 | -1 | 1 | 0.48 |
|  | 359 | 294 | 22 | 2 | 0.51 | 1278 | 1092 | 17 | 2 | 0.37 |
| Short-FFQ in JPHC-NEXT ^(65)^ | 266 | 329 | -19 | 2 | 0.34 | 922 | 1275 | -28 | 2 | 0.47 |
|  | 255 | 296 | -14 | 2 | 0.45 | 810 | 1097 | -26 | 2 | 0.54 |
| Short version FFQ ^(68)^ | 247 | 233 | 6 | 3 | 0.52 | 952 | 909 | 5 | 3 | 0.45 |
| Maruyama FFQ ^(69)^ | 300 | 320 | -6 | 3 | 0.59 | - | - | - | - | - |

| FFQ | Iron (mg/d) | | | | | Zinc (mg/d) | | | | |
| --- | --- | --- | --- | --- | --- | --- | --- | --- | --- | --- |
|  | Group level (mean or median) | | | | Correlation coefficient | Group level (mean or median) | | | | Correlation coefficient |
|  | Mean [median] (FFQ) | Mean [median] (DR) | Difference (%): FFQ−DR)/DR*100 | Statistics: 1: No difference 2: Difference 3: Not tested |  | Mean [median] (FFQ) | Mean [median] (DR) | Difference (%): FFQ−DR)/DR*100 | Statistics: 1: No difference 2: Difference 3: Not tested |  |
| 47-item FFQ ^(38)^ | 7.7 | 9.8 | -21 | 2 | 0.58 | - | - | - | - | - |
|  | 7.7 | 8.9 | -14 | 2 | 0.44 | - | - | - | - | - |
| 47-item FFQ ^(41)^ | - | - | - | - | - | - | - | - | - | - |
|  | - | - | - | - | - | - | - | - | - | - |
| DHQ ^(43)^ | 7.2 | 9.1 | -20 | 2 | 0.48 | 8.8 | 9.8 | -10 | 2 | 0.36 |
|  | 7.1 | 8.1 | -13 | 2 | 0.67 | 7.9 | 8.1 | -3 | 2 | 0.60 |
| BDHQ ^(44)^ | 8.6 | 9.1 | -5 | 2 | 0.61 | 9.1 | 9.8 | -7 | 2 | 0.48 |
|  | 7.9 | 8.1 | -2 | 1 | 0.57 | 8.1 | 8.1 | 0 | 1 | 0.39 |
| MDHQ ^(51)^ | 5.96 | 7.70 | -23 | 2 | 0.52 | 7.23 | 9.20 | -21 | 2 | 0.42 |
|  | 5.41 | 6.72 | -20 | 2 | 0.61 | 6.10 | 7.56 | -19 | 2 | 0.43 |
| FFQ in JACC ^(52)^ | 8.5 | 13.1 | -35 | 3 | 0.35 | - | - | - | - | - |
|  | 8.0 | 12.0 | -33 | 3 | 0.47 | - | - | - | - | - |
| JPHC FFQ at baseline ^(55)^ | 8.3 | 12.8 | -35 | 3 | 0.31 | - | - | - | - | - |
|  | 7.2 | 11.2 | -36 | 3 | 0.30 | - | - | - | - | - |
| JPHC_5y ^(56)^ | 12.2 | 12.9 | -5 | 3 | 0.49 | - | - | - | - | - |
|  | 12.1 | 11.3 | 7 | 3 | 0.33 | - | - | - | - | - |
| FFQ in JPHC-NEXT ^(65)^ | 9.7 | 9.5 | 2 | 1 | 0.53 | 9.3 | 9.6 | -3 | 1 | 0.44 |
|  | 9.7 | 8.8 | 10 | 2 | 0.57 | 8.7 | 7.9 | 11 | 2 | 0.27 |
| Short-FFQ in JPHC-NEXT ^(65)^ | 7.6 | 9.7 | -21 | 2 | 0.56 | 7.2 | 9.7 | -26 | 2 | 0.40 |
|  | 7.2 | 8.9 | -19 | 2 | 0.63 | 6.1 | 7.9 | -23 | 2 | 0.38 |
| Short version FFQ ^(68)^ | 8.1 | 7.5 | 8 | 3 | 0.38 | 7.6 | 7.4 | 3 | 3 | 0.31 |
| Maruyama FFQ ^(69)^ | - | - | - | - | - | - | - | - | - | - |

| FFQ | Copper (mg/d) | | | | |
| --- | --- | --- | --- | --- | --- |
|  | Group level (mean or median) | | | | Correlation coefficient |
|  | Mean [median] (FFQ) | Mean [median] (DR) | Difference (%): FFQ−DR)/DR*100 | Statistics: 1: No difference 2: Difference 3: Not tested |  |
| 47-item FFQ ^(38)^ | - | - | - | - | - |
|  | - | - | - | - | - |
| 47-item FFQ ^(41)^ | - | - | - | - | - |
|  | - | - | - | - | - |
| DHQ ^(43)^ | 1.2 | 1.4 | -12 | 2 | 0.61 |
|  | 1.1 | 1.2 | -4 | 2 | 0.66 |
| BDHQ ^(44)^ | 1.4 | 1.4 | -2 | 1 | 0.59 |
|  | 1.2 | 1.2 | 2 | 1 | 0.61 |
| MDHQ ^(51)^ | 0.99 | 1.18 | -16 | 2 | 0.53 |
|  | 0.84 | 1.00 | -16 | 2 | 0.58 |
| FFQ in JACC ^(52)^ | - | - | - | - | - |
|  | - | - | - | - | - |
| JPHC FFQ at baseline ^(55)^ | - | - | - | - | - |
|  | - | - | - | - | - |
| JPHC_5y ^(56)^ | - | - | - | - | - |
|  | - | - | - | - | - |
| FFQ in JPHC-NEXT ^(65)^ | 1.4 | 1.4 | -2 | 1 | 0.64 |
|  | 1.4 | 1.3 | 10 | 2 | 0.49 |
| Short-FFQ in JPHC-NEXT ^(65)^ | 1.1 | 1.5 | -28 | 2 | 0.54 |
|  | 1.0 | 1.3 | -25 | 2 | 0.65 |
| Short version FFQ ^(68)^ | 1.2 | 1.1 | 5 | 3 | 0.41 |
| Maruyama FFQ ^(69)^ | - | - | - | - | - |

FFQ, Food frequency questionnaire; DR, Dietary record; 47-item FFQ, 47-item short food frequency questionnaire; DHQ, Self-administered diet history questionnaire; BDHQ, Brief-type self-administered diet history questionnaire; MDHQ, Meal-based Diet History Questionnaire; JPHC FFQ at baseline, 44-item food frequency questionnaire; JPHC_5y, JPHC FFQ at 5-year follow-up; FFQ in JPHC-NeXT, Long-FFQ in JPHC-NeXT; Short-FFQ in JPHC-NeXT, 66-item food frequency questionnaire for the Japan Public Health Centre-based prospective Study for the Next Generation (JPHC-NEXT) follow-up survey; Short version FFQ, Short version of the Shizuoka Prefecture version of the Food Intake Frequency Questionnaire; Maruyama FFQ, Maruyama food frequency questionnaire.

Supplementary Table 3. The results of the validity assessment of dietary questionnaires to estimate food group intake among Japanese adults in main articles

| FFQ | Participant characteristics | | | Energy-adjustment methods in mean value calculation 1: Crude 2: Density method 3: Residual method | Statistics: Correlations  1: Correlation 2: Adjusted correlation (energy) 3: Deattenuated or intraclass correlation | Type of correlation coefficient P: Pearson S: Spearman |  |
| --- | --- | --- | --- | --- | --- | --- | --- |
|  |  |  |  |  |  |  |  |
|  |  |  |  |  |  |  |  |
|  | Sex M: Male F: Female | Age (years) | n |  |  |  |  |
| 47-item FFQ ^(41)^ | M | 35-69 | 143 | 3 | 3 | S |  |
|  | F | 35-69 | 145 | 3 | 3 | S |  |
| DHQ ^(43)^ | M | 32-76 | 92 | 2 | 2 | S |  |
|  | F | 31-69 | 92 | 2 | 2 | S |  |
| BDHQ ^(43)^ | M | 32-76 | 92 | 2 | 2 | S |  |
|  | F | 31-69 | 92 | 2 | 2 | S |  |
| MDHQ ^(49)^ | M | 30-69 | 111 | 1 | 1 | S |  |
|  | F | 30-69 | 111 | 1 | 1 | S |  |
| FFQ in JACC ^(52)^ | M | 45–77 | 55 | 1 | 3 | S |  |
|  | F | 47–76 | 58 | 1 | 3 | S |  |
| JPHC FFQ at baseline ^(55)^ | M | 40-59 | 94 | 3 | 2 | S |  |
|  | F | 40-59 | 107 | 3 | 2 | S |  |
| JPHC_5y ^(57)^ | M | - | 102 | 3 | 2 | S |  |
|  | F | - | 113 | 3 | 2 | S |  |
| FFQ in JPHC-NEXT ^(65)^ | M | 40-74 | 98 | 1 | 3 | S |  |
|  | F | 40-74 | 142 | 1 | 3 | S |  |
| Short-FFQ in JPHC-NEXT ^(65)^ | M | 40-74 | 92 | 1 | 3 | S |  |
|  | F | 40-74 | 136 | 1 | 3 | S |  |
|  |  |  |  |  |  |  |  |
| Short version FFQ ^(68)^ | MF | mean: 55.7 | 491 | 2 | 2 | S |  |
| Maruyama FFQ ^(69)^ | MF | 47-78 | 58 | 3 | 2 | S |  |

| FFQ | Grains | | | | | | | | | |
| --- | --- | --- | --- | --- | --- | --- | --- | --- | --- | --- |
|  | Total (g/d) | | | | | Rice (g/d) | | | | |
|  | Group level (mean or median) | | | | Correlation coefficient | Group level (mean or median) | | | | Correlation coefficient |
|  | Mean [median] (FFQ) | Mean [median] (DR) | Difference (%): FFQ−DR)/DR*100 | Statistics: 1: No difference 2: Difference 3: Not tested |  | Mean [median] (FFQ) | Mean [median] (DR) | Difference (%): FFQ−DR)/DR*100 | Statistics: 1: No difference 2: Difference 3: Not tested |  |
| 47-item FFQ ^(41)^ | - | - | - | - | - | 447.0 | 377.0 | 19 | 3 | 0.67 |
|  | - | - | - | - | - | 273.0 | 234.0 | 17 | 3 | 0.61 |
| DHQ ^(43)^ | 551.9 | 550.3 | 0.3 | 1 | 0.58 | 456.8 | 398.6 | 15 | 2 | 0.61 |
|  | 494.5 | 512.6 | -3.5 | 1 | 0.45 | 368.4 | 358.2 | 3 | 1 | 0.63 |
| BDHQ ^(43)^ | 561.1 | 550.3 | 2.0 | 1 | 0.60 | 425.5 | 398.6 | 7 | 2 | 0.61 |
|  | 516.5 | 512.6 | 0.8 | 1 | 0.44 | 404.0 | 358.2 | 13 | 2 | 0.54 |
| MDHQ ^(49)^ | - | - | - | - | - | 315 | 313 | 1 | 1 | 0.54 |
|  | - | - | - | - | - | 218 | 204 | 7 | 1 | 0.61 |
| FFQ in JACC ^(52)^ | - | - | - | - | - | 726.0 | 563.4 | 29 | 3 | 0.16 |
|  | - | - | - | - | - | 399.0 | 356.4 | 12 | 3 | 0.65 |
| JPHC FFQ at baseline ^(55)^ | 342.7 | 311.1 | 10 | 3 | 0.36 | - | - | - | - | - |
|  | 316.7 | 220.4 | 44 | 3 | 0.27 | - | - | - | - | - |
| JPHC_5y ^(57)^ | 349.0 | 313.0 | 11 | 3 | 0.42 | - | - | - | - | - |
|  | 294.0 | 220.0 | 33 | 3 | 0.27 | - | - | - | - | - |
| FFQ in JPHC-NEXT ^(65)^ | 637.0 | 508.0 | 25 | 3 | 0.60 | 460.0 | 394.0 | 17 | 3 | 0.58 |
|  | 472.0 | 348.0 | 35 | 3 | 0.31 | 326.0 | 259.0 | 26 | 3 | 0.48 |
| Short-FFQ in JPHC-NEXT ^(65)^ | 538.0 | 508.0 | 6 | 3 | 0.57 | 449.0 | 394.0 | 14 | 3 | 0.57 |
|  | 403.0 | 348.0 | 16 | 3 | 0.45 | 325.0 | 261.0 | 24 | 3 | 0.57 |
|  |  |  |  |  |  |  |  |  |  |  |
| Short version FFQ ^(68)^ | 485.5 | 460.7 | 5 | 3 | 0.35 | - | - | - | - | - |
| Maruyama FFQ ^(69)^ | 415.5 | 453.1 | -8 | 3 | 0.75 | - | - | - | - | - |

| FFQ | Potato | | | | | Sugar and sweeteners | | | | |
| --- | --- | --- | --- | --- | --- | --- | --- | --- | --- | --- |
|  | Total (g/d) | | | | | Total (g/d) | | | | |
|  | Group level (mean or median) | | | | Correlation coefficient | Group level (mean or median) | | | | Correlation coefficient |
|  | Mean [median] (FFQ) | Mean [median] (DR) | Difference (%): FFQ−DR)/DR*100 | Statistics: 1: No difference 2: Difference 3: Not tested |  | Mean [median] (FFQ) | Mean [median] (DR) | Difference (%): FFQ−DR)/DR*100 | Statistics: 1: No difference 2: Difference 3: Not tested |  |
| 47-item FFQ ^(41)^ | 16.0 | 45.0 | -64 | 3 | 0.17 | - | - | - | - | - |
|  | 19.0 | 38.0 | -50 | 3 | 0.41 | - | - | - | - | - |
| DHQ ^(43)^ | 32.9 | 69.9 | -53 | 2 | 0.30 | 12.9 | 9.7 | 33.0 | 2 | 0.41 |
|  | 54.3 | 76.0 | -29 | 2 | 0.13 | 15.4 | 11.3 | 36.3 | 2 | 0.46 |
| BDHQ ^(43)^ | 61.5 | 69.9 | -12 | 1 | 0.21 | 6.6 | 9.7 | -32.0 | 2 | 0.30 |
|  | 77.7 | 76.0 | 2 | 1 | 0.17 | 6.9 | 11.3 | -38.9 | 1 | 0.34 |
| MDHQ ^(49)^ | 22 | 28 | -21 | 2 | 0.25 | - | - | - | - | - |
|  | 24 | 27 | -11 | 1 | 0.21 | - | - | - | - | - |
| FFQ in JACC ^(52)^ | 23.0 | 47.9 | -52 | 3 | 0.53 | - | - | - | - | - |
|  | 31.5 | 44.3 | -29 | 3 | 0.08 | - | - | - | - | - |
| JPHC FFQ at baseline ^(55)^ | 19.5 | 48.6 | 0 | 3 | 0.24 | - | - | - | - | - |
|  | 21.1 | 48.1 | -60 | 3 | 0.19 | - | - |  | - | - |
| JPHC_5y ^(57)^ | 32.0 | 51.0 | -37 | 3 | 0.33 | - | - | - | - | - |
|  | 38.0 | 49.0 | -22 | 3 | 0.20 | - | - | - | - | - |
| FFQ in JPHC-NEXT ^(65)^ | 37.0 | 45.0 | -56 | 3 | 0.28 | 0 | 6.0 | N/A | 3 | 0.34 |
|  | 44.0 | 43.0 | -17 | 3 | 0.45 | 1.0 | 6.0 | -91 | 3 | 0.23 |
| Short-FFQ in JPHC-NEXT ^(65)^ | - | 45.0 | 2 | - | - | - | 6.0 | - | - | - |
|  | - | 44.0 | - | - | - | - | 6.0 | - | - | - |
|  |  |  |  |  |  |  |  |  |  |  |
| Short version FFQ ^(68)^ | 42.6 | 65.6 | -35 | 3 | 0.14 | 5.4 | 6.3 | -14 | 3 | 0.21 |
| Maruyama FFQ ^(69)^ | - | - | - | - | - | - | - | - | - | - |

| FFQ | Beans | | | | | Seeds and nuts | | | | |
| --- | --- | --- | --- | --- | --- | --- | --- | --- | --- | --- |
|  | Total (g/d) | | | | | Total (g/d) | | | | |
|  | Group level (mean or median) | | | | Correlation coefficient | Group level (mean or median) | | | | Correlation coefficient |
|  | Mean [median] (FFQ) | Mean [median] (DR) | Difference (%): FFQ−DR)/DR*100 | Statistics: 1: No difference 2: Difference 3: Not tested |  | Mean [median] (FFQ) | Mean [median] (DR) | Difference (%): FFQ−DR)/DR*100 | Statistics: 1: No difference 2: Difference 3: Not tested |  |
| 47-item FFQ ^(41)^ | - | - | - | - | - | - | - | - | - | - |
|  | - | - | - | - | - | - | - | - | - | - |
| DHQ ^(43)^ | 54.8 | 62.5 | -12.3 | 2 | 0.49 | 0 | 2.0 | N/A | 2 | 0.15 |
|  | 70.7 | 83.1 | -14.9 | 2 | 0.42 | 0 | 2.5 | N/A | 2 | 0.20 |
| BDHQ ^(43)^ | 95.3 | 62.5 | 52.5 | 2 | 0.39 | - | - | - | - | - |
|  | 111.6 | 83.1 | 34.3 | 2 | 0.41 | - | - | - | - | - |
| MDHQ ^(49)^ | 30 | 39 | -23.0 | 2 | 0.39 | - | - | - | - | - |
|  | 37 | 44 | -16.0 | 2 | 0.46 | - | - | - | - | - |
| FFQ in JACC ^(52)^ | - | - | - | - | - | - | - | - | - | - |
|  | - | - | - | - | - | - | - | - | - | - |
| JPHC FFQ at baseline ^(55)^ | 61.2 | 86.9 | -29.6 | 3 | 0.39 | - | - | - | - | - |
|  | 57.9 | 74.1 | -21.9 | 3 | 0.43 | - | - | - | - | - |
| JPHC_5y ^(57)^ | 78.0 | 87.0 | -11.0 | 3 | 0.53 | 2.0 | 2.0 | -1 | 3 | 0.26 |
|  | 74.0 | 74.0 | 0.0 | 3 | 0.49 | 3.0 | 3.0 | -2 | 3 | 0.15 |
| FFQ in JPHC-NEXT ^(65)^ | 78.0 | 70.0 | 11.4 | 3 | 0.62 | - | - | - | - | - |
|  | 77.0 | 70.0 | 10.0 | 3 | 0.61 | - | - | - | - | - |
| Short-FFQ in JPHC-NEXT ^(65)^ | 69.0 | 72.0 | -4.2 | 3 | 0.63 | - | - | - | - | - |
|  | 64.0 | 71.0 | -9.9 | 3 | 0.59 | - | - | - | - | - |
|  |  |  |  |  |  |  |  |  |  |  |
| Short version FFQ ^(68)^ | 71.0 | 50.1 | 42.0 | 3 | 0.30 | 2.7 | 2 | 50.0 | 3 | 0.13 |
| Maruyama FFQ ^(69)^ | 130.0 | 70.9 | 83.4 | 3 | 0.54 | 5.5 | 4.3 | 28 | 3 | 0.44 |

| FFQ | Vegetables | | | | | Fruits | | | | |
| --- | --- | --- | --- | --- | --- | --- | --- | --- | --- | --- |
|  | Total (g/d) | | | | | Total (g/d) | | | | |
|  | Group level (mean or median) | | | | Correlation coefficient | Group level (mean or median) | | | | Correlation coefficient |
|  | Mean [median] (FFQ) | Mean [median] (DR) | Difference (%): FFQ−DR)/DR*100 | Statistics: 1: No difference 2: Difference 3: Not tested |  | Mean [median] (FFQ) | Mean [median] (DR) | Difference (%): FFQ−DR)/DR*100 | Statistics: 1: No difference 2: Difference 3: Not tested |  |
| 47-item FFQ ^(41)^ | - | - | - | - | - | 47.0 | 129.0 | -64 | 3 | 0.62 |
|  | - | - | - | - | - | 64.0 | 117.0 | -45 | 3 | 0.58 |
| DHQ ^(43)^ | 223.5 | 301.5 | -26 | 2 | 0.40 | 119.5 | 90.8 | 32 | 2 | 0.68 |
|  | 306.2 | 360.6 | -15 | 2 | 0.56 | 174.6 | 141.4 | 23 | 2 | 0.40 |
| BDHQ ^(43)^ | 326.0 | 301.5 | 8 | 1 | 0.51 | 112.4 | 90.8 | 24 | 2 | 0.55 |
|  | 422.5 | 360.6 | 17 | 2 | 0.55 | 169.5 | 141.4 | 20 | 2 | 0.41 |
| MDHQ ^(49)^ | 173 | 216 | -20 | 2 | 0.46 | 24 | 34 | -29 | 1 | 0.64 |
|  | 167 | 190 | -12 | 2 | 0.37 | 47 | 50 | -6 | 1 | 0.55 |
| FFQ in JACC ^(52)^ | - | - | - | - | - | - | - | - | - | - |
|  | - | - | - | - | - | - | - | - | - | - |
| JPHC FFQ at baseline ^(55)^ | 184.1 | 309.7 | -41 | 3 | 0.27 | 93.1 | 121.2 | -23 | 3 | 0.55 |
|  | 176.3 | 297.6 | -41 | 3 | 0.31 | 139.8 | 156.3 | -11 | 3 | 0.35 |
| JPHC_5y ^(57)^ | 250.0 | 341.0 | -21 | 3 | 0.22 | 204.0 | 120.0 | 69 | 3 | 0.41 |
|  | 284.0 | 300.0 | -6 | 3 | 0.32 | 264.0 | 156.0 | 69 | 3 | 0.23 |
| FFQ in JPHC-NEXT ^(65)^ | 277.0 | 358.0 | -23 | 3 | 0.59 | 147.0 | 94.0 | 56 | 3 | 0.75 |
|  | 359.0 | 344.0 | 4 | 3 | 0.59 | 260.0 | 139.0 | 88 | 3 | 0.56 |
| Short-FFQ in JPHC-NEXT ^(65)^ | 138.0 | 365.0 | -62 | 3 | 0.49 | 98.0 | 96.0 | 2 | 3 | 0.60 |
|  | 188.0 | 350.0 | -46 | 3 | 0.42 | 150.0 | 139.0 | 7 | 3 | 0.50 |
|  |  |  |  |  |  |  |  |  |  |  |
| Short version FFQ ^(68)^ | 222.7 | 285.0 | -22 | 3 | 0.39 | 137.2 | 102.7 | 34 | 3 | 0.39 |
| Maruyama FFQ ^(69)^ | 214.0 | 289.6 | -26 | 3 | 0.34 | 158.1 | 154.0 | 3 | 3 | 0.71 |

| FFQ | Mushrooms | | | | | Seaweed | | | | |
| --- | --- | --- | --- | --- | --- | --- | --- | --- | --- | --- |
|  | Total (g/d) | | | | | Total (g/d) | | | | |
|  | Group level (mean or median) | | | | Correlation coefficient | Group level (mean or median) | | | | Correlation coefficient |
|  | Mean [median] (FFQ) | Mean [median] (DR) | Difference (%): FFQ−DR)/DR*100 | Statistics: 1: No difference 2: Difference 3: Not tested |  | Mean [median] (FFQ) | Mean [median] (DR) | Difference (%): FFQ−DR)/DR*100 | Statistics: 1: No difference 2: Difference 3: Not tested |  |
| 47-item FFQ ^(41)^ | 7.0 | 14.0 | -50 | 3 | 0.26 | 1.0 | 13.0 | -92 | 3 | 0.35 |
|  | 11.0 | 16.0 | -31 | 3 | 0.26 | 2.0 | 11.0 | -82 | 3 | 0.23 |
| DHQ ^(43)^ | 10.0 | 10.6 | -6 | 1 | 0.41 | 9.7 | 11.9 | -18 | 1 | 0.08 |
|  | 17.6 | 13.0 | 35 | 2 | 0.34 | 16.0 | 14.4 | 11 | 1 | 0.14 |
| BDHQ ^(43)^ | 13.0 | 10.6 | 23 | 2 | 0.59 | 12.0 | 11.9 | 1 | 1 | 0.32 |
|  | 16.6 | 13.0 | 28 | 2 | 0.56 | 17.7 | 14.4 | 23 | 1 | 0.17 |
| MDHQ ^(49)^ | - | - | - | - | - | - | - | - | - | - |
|  | - | - | - | - | - | - | - | - | - | - |
| FFQ in JACC ^(52)^ | 5.8 | 7.6 | -24 | 3 | 0.32 | 4.8 | 12.2 | -61 | 3 | 0.44 |
|  | 3.4 | 6.7 | -49 | 3 | 0.55 | 6.4 | 12.1 | -47 | 3 | 0.00 |
| JPHC FFQ at baseline ^(55)^ | 3.0 | 8.6 | -65 | 3 | 0.30 | 2.0 | 7.8 | -74 | 3 | 0.21 |
|  | 3.5 | 7.9 | -56 | 3 | 0.28 | 1.7 | 7.1 | -76 | 3 | 0.19 |
| JPHC_5y ^(57)^ | 10.0 | 9.0 | 20 | 3 | 0.44 | 12.0 | 8.0 | 53 | 3 | 0.08 |
|  | 12.0 | 8.0 | 53 | 3 | 0.38 | 13.0 | 7.0 | 78 | 3 | 0.06 |
| FFQ in JPHC-NEXT ^(65)^ | 11.0 | 21.0 | -49 | 3 | 0.26 | 8.0 | 10.0 | -22 | 3 | 0.25 |
|  | 16.0 | 18.0 | -12 | 3 | 0.41 | 9.0 | 9.0 | 0 | 3 | 0.32 |
| Short-FFQ in JPHC-NEXT ^(65)^ | 5.0 | 21.0 | -78 | 3 | 0.29 | 7.0 | 10.0 | -27 | 3 | 0.22 |
|  | 6.0 | 18.0 | -68 | 3 | 0.27 | 8.0 | 9.0 | -13 | 3 | 0.26 |
|  |  |  |  |  |  |  |  |  |  |  |
| Short version FFQ ^(68)^ | 13.9 | 13.8 | 1 | 3 | 0.80 | 11.0 | 11.8 | -7 | 3 | 0.16 |
| Maruyama FFQ ^(69)^ | - | - | - | - | - | 8.1 | 2 | -76 | 3 | 0.49 |

| FFQ | Fish and shellfish | | | | | | | | | | | Meat | | | | | | | | | |  |
| --- | --- | --- | --- | --- | --- | --- | --- | --- | --- | --- | --- | --- | --- | --- | --- | --- | --- | --- | --- | --- | --- | --- |
|  | Total (g/d) | | | | | | | | | | | Total (g/d) | | | | | | | | | |  |
|  | Group level (mean or median) | | | | | | | | Correlation coefficient | | | Group level (mean or median) | | | | | | | |  |  |  |
|  | Mean [median] (FFQ) | | Mean [median] (DR) | | Difference (%): FFQ−DR)/DR*100 | | Statistics: 1: No difference 2: Difference 3: Not tested | | |  | | Mean [median] (FFQ) | Mean [median] (DR) | | Difference (%): FFQ−DR)/DR*100 | | | Statistics: 1: No difference 2: Difference 3: Not tested | Correlation coefficien | | |  |
| 47-item FFQ ^(41)^ | 46.0 | | 80.0 | | -43 | | 3 | | | 0.38 | | 39.0 | 106.0 | | -63 | | | 3 | 0.41 | | |  |
|  | 46.0 | | 62.0 | | -26 | | 3 | | | 0.58 | | 45.0 | 73.0 | | -38 | | | 3 | 0.41 | | |  |
| DHQ ^(43)^ | 86.3 | | 98.8 | | -13 | | 2 | | | 0.37 | | 72.0 | 75.8 | | -5 | | | 1 | 0.49 | | |  |
|  | 86.4 | | 99.7 | | -13 | | 1 | | | 0.54 | | 66.8 | 68.6 | | -3 | | | 1 | 0.66 | | |  |
| BDHQ ^(43)^ | 86.2 | | 98.8 | | -13 | | 1 | | | 0.29 | | 70.4 | 75.8 | | -7 | | | 2 | 0.44 | | |  |
|  | 105.3 | | 99.7 | | 6 | | 1 | | | 0.41 | | 78.2 | 68.6 | | 14 | | | 1 | 0.63 | | |  |
| MDHQ ^(49)^ | 39 | | 55 | | -29 | | 2 | | | 0.3 | | 72 | 112 | | -36 | | | 2 | 0.21 | | |  |
|  | 29 | | 45 | | -36 | | 2 | | | 0.41 | | 58 | 81 | | -28 | | | 2 | 0.31 | | |  |
| FFQ in JACC ^(52)^ | - | | - | | - | | - | | | - | | - | - | | - | | | - | - | | |  |
|  | - | | - | | - | | - | | | - | | - | - | | - | | | - | - | | |  |
| JPHC FFQ at baseline ^(55)^ | 48.4 | | 137.1 | | -65 | | 3 | | | 0.37 | | 41.9 | 76.4 | | -45 | | | 3 | 0.18 | | |  |
|  | 39.7 | | 107.5 | | -63 | | 3 | | | 0.32 | | 33.3 | 58.9 | | -43 | | | 3 | 0.26 | | |  |
| JPHC_5y ^(57)^ | 114.0 | | 136.0 | | -16 | | 3 | | | 0.32 | | 71.0 | 76.0 | | -7 | | | 3 | 0.50 | | |  |
|  | 105.0 | | 106.0 | | -1 | | 3 | | | 0.32 | | 58.0 | 60.0 | | -3 | | | 3 | 0.45 | | |  |
| FFQ in JPHC-NEXT ^(65)^ | 76.0 | | 108.0 | | -30 | | 3 | | | 0.30 | | 71.0 | 87.0 | | -19 | | | 3 | 0.44 | | |  |
|  | 76.0 | | 82.0 | | -6 | | 3 | | | 0.47 | | 56.0 | 61.0 | | -8 | | | 3 | 0.29 | | |  |
| Short-FFQ in JPHC-NEXT ^(65)^ | 60.0 | | 109.0 | | -44 | | 3 | | | 0.30 | | 56.0 | 89.0 | | -37 | | | 3 | 0.38 | | |  |
|  | 60.0 | | 82.0 | | -26 | | 3 | | | 0.56 | | 43.0 | 61.0 | | -30 | | | 3 | 0.44 | | |  |
|  |  | |  | |  | |  | | |  | |  |  | |  | | |  |  | | |  |
| Short version FFQ ^(68)^ | 74.2 | | 84.9 | | -13 | | 3 | | | 0.32 | | 71.6 | 77.6 | | -8 | | | 3 | 0.29 | | |  |
| Maruyama FFQ ^(69)^ | 92.7 | | 97.4 | | -5 | | 3 | | | 0.30 | | 45.4 | 60.0 | | -24 | | | 3 | 0.24 | | |  |
| FFQ | | Meat | | | | | | | | | | | | | | | | | | | | |
|  |  | Beef (g/d) | | | | | | | | | | | | Pork (g/d) | | | | | | | | |
|  |  | Group level (mean or median) | | | | | | | | | Correlation coefficient | | | Group level (mean or median) | | | | | | | | Correlation coefficient |
|  |  | Mean [median] (FFQ) | | Mean [median] (DR) | | Difference (%): FFQ−DR)/DR*100 | | Statistics: 1: No difference 2: Difference 3: Not tested | | |  |  |  | Mean [median] (FFQ) | | Mean [median] (DR) | Difference (%): FFQ−DR)/DR*100 | | | Statistics: 1: No difference 2: Difference 3: Not tested | |  |
| 47-item FFQ ^(41)^ | | - | | - | | - | | - | | | - | | | - | | - | - | | | - | | - |
|  | | - | | - | | - | | - | | | - | | | - | | - | - | | | - | | - |
| DHQ ^(43)^ | | - | | - | | - | | - | | | - | | | - | | - | - | | | - | | - |
|  | | - | | - | | - | | - | | | - | | | - | | - | - | | | - | | - |
| BDHQ ^(43)^ | | - | | - | | - | | - | | | - | | | - | | - | - | | | - | | - |
|  | | - | | - | | - | | - | | | - | | | - | | - | - | | | - | | - |
| MDHQ ^(49)^ | | - | | - | | - | | - | | | - | | | - | | - | - | | | - | | - |
|  | | - | | - | | - | | - | | | - | | | - | | - | - | | | - | | - |
| FFQ in JACC ^(52)^ | | 3.8 | | 0.8 | | 375 | | 3 | | | 0.36 | | | 10.4 | | 16.4 | -37 | | | 3 | | 0.20 |
|  | | 2.7 | | 0.8 | | 238 | | 3 | | | 0.13 | | | 8.7 | | 10.8 | -19 | | | 3 | | 0.75 |
| JPHC FFQ at baseline ^(55)^ | | - | | - | | - | | - | | | - | | | - | | - | - | | | - | | - |
|  | | - | | - | | - | | - | | | - | | | - | | - | - | | | - | | - |
| JPHC_5y ^(57)^ | | - | | - | | - | | - | | | - | | | - | | - | - | | | - | | - |
|  | | - | | - | | - | | - | | | - | | | - | | - | - | | | - | | - |
| FFQ in JPHC-NEXT ^(65)^ | | - | | - | | - | | - | | | - | | | - | | - | - | | | - | | - |
|  | | - | | - | | - | | - | | | - | | | - | | - | - | | | - | | - |
| Short-FFQ in JPHC-NEXT ^(65)^ | | - | | - | | - | | - | | | - | | | - | | - | - | | | - | | - |
|  | | - | | - | | - | | - | | | - | | | - | | - | - | | | - | | - |
|  | |  | |  | |  | |  | | |  | | |  | |  |  | | |  | |  |
| Short version FFQ ^(68)^ | | - | | - | | - | | - | | | - | | | - | | - | - | | | - | | - |
| Maruyama FFQ ^(69)^ | | - | | - | | - | | - | | | - | | | - | | - | - | | | - | | - |

| FFQ | Meat | | | | | | | | | |
| --- | --- | --- | --- | --- | --- | --- | --- | --- | --- | --- |
|  | Ham and sausages (g/d) | | | | | Meat-processed | | | | |
|  | Group level (mean or median) | | | | Correlation coefficient | Group level (mean or median) | | | | Correlation coefficient |
|  | Mean [median] (FFQ) | Mean [median] (DR) | Difference (%): FFQ−DR)/DR*100 | Statistics: 1: No difference 2: Difference 3: Not tested |  | Mean [median] (FFQ) | Mean [median] (DR) | Difference (%): FFQ−DR)/DR*100 | Statistics: 1: No difference 2: Difference 3: Not tested |  |
| 47-item FFQ ^(41)^ | - | - | - | - | - | - | - | - | - | - |
|  | - | - | - | - | - | - | - | - | - | - |
| DHQ ^(43)^ | - | - | - | - | - | - | - | - | - | - |
|  | - | - | - | - | - | - | - | - | - | - |
| BDHQ ^(43)^ | - | - | - | - | - | - | - | - | - | - |
|  | - | - | - | - | - | - | - | - | - | - |
| MDHQ ^(49)^ | - | - | - | - | - | - | - | - | - | - |
|  | - | - | - | - | - | - | - | - | - | - |
| FFQ in JACC ^(52)^ | 3.1 | 6.2 | -50 | 3 | 0.37 | - | - | - | - | - |
|  | 3.2 | 4.9 | -35 | 3 | 0.43 | - | - | - | - | - |
| JPHC FFQ at baseline ^(55)^ | - | - | - | - | - | - | - | - | - | - |
|  | - | - | - | - | - | - | - | - | - | - |
| JPHC_5y ^(57)^ | - | - | - | - | - | - | - | - | - | - |
|  | - | - | - | - | - | - | - | - | - | - |
| FFQ in JPHC-NEXT ^(65)^ | - | - | - | - | - | 10.0 | 17.0 | -40 | 3 | 0.34 |
|  | - | - | - | - | - | 9.0 | 12.0 | -20 | 3 | 0.60 |
| Short-FFQ in JPHC-NEXT ^(65)^ | - | - | - | - | - | - | 18.0 | - | - | - |
|  | - | - | - | - | - | - | 11.0 | - | - | - |
|  |  |  |  |  |  |  |  |  |  |  |
| Short version FFQ ^(68)^ | - | - | - | - | - | - | - | - | - | - |
| Maruyama FFQ ^(69)^ | - | - | - | - | - | - | - | - | - | - |

| FFQ | Meat | | | | | Egg | | | | |
| --- | --- | --- | --- | --- | --- | --- | --- | --- | --- | --- |
|  | Meat-red meat | | | | | Total (g/d) | | | | |
|  | Group level (mean or median) | | | | Correlation coefficient | Group level (mean or median) | | | | Correlation coefficient |
|  | Mean [median] (FFQ) | Mean [median] (DR) | Difference (%): FFQ−DR)/DR*100 | Statistics: 1: No difference 2: Difference 3: Not tested |  | Mean [median] (FFQ) | Mean [median] (DR) | Difference (%): FFQ−DR)/DR*100 | Statistics: 1: No difference 2: Difference 3: Not tested |  |
| 47-item FFQ ^(41)^ | - | - | - | - | - | 20.0 | 44.0 | -55 | 3 | 0.48 |
|  | - | - | - | - | - | 23.0 | 36.0 | -36 | 3 | 0.47 |
| DHQ ^(43)^ | - | - | - | - | - | 40.6 | 44.2 | -8 | 1 | 0.52 |
|  | - | - | - | - | - | 43.5 | 45.3 | -4 | 1 | 0.38 |
| BDHQ ^(43)^ | - | - | - | - | - | 47.4 | 44.2 | 7 | 1 | 0.55 |
|  | - | - | - | - | - | 47.1 | 45.3 | 4 | 1 | 0.32 |
| MDHQ ^(49)^ | - | - | - | - | - | 28 | 44 | -36 | 2 | 0.66 |
|  | - | - | - | - | - | 26 | 32 | -19 | 2 | 0.49 |
| FFQ in JACC ^(52)^ | - | - | - | - | - | 41.4 | 44.8 | -8 | 3 | 0.53 |
|  | - | - | - | - | - | 37.0 | 36.6 | 1 | 3 | 0.58 |
| JPHC FFQ at baseline ^(55)^ | - | - | - | - | - | 30.8 | 39.3 | -22 | 3 | 0.25 |
|  | - | - | - | - | - | 28.9 | 33.6 | -14 | 3 | 0.28 |
| JPHC_5y ^(57)^ | - | - | - | - | - | 33.0 | 39.0 | -16 | 3 | 0.25 |
|  | - | - | - | - | - | 31.0 | 33.0 | -7 | 3 | 0.42 |
| FFQ in JPHC-NEXT ^(65)^ | 40.0 | 46.0 | -13 | 3 | 0.43 | 44.0 | 40.0 | 10 | 3 | 0.56 |
|  | 30.0 | 32.0 | -6 | 3 | 0.20 | 37.0 | 32.0 | 14 | 3 | 0.48 |
| Short-FFQ in JPHC-NEXT ^(65)^ | 46.0 | 47.0 | -2 | 3 | 0.40 | 44.0 | 40.0 | 8 | 3 | 0.52 |
|  | 36.0 | 32.0 | 14 | 3 | 0.34 | 27.0 | 32.0 | -16 | 3 | 0.50 |
|  |  |  |  |  |  |  |  |  |  |  |
| Short version FFQ ^(68)^ | - | - | - | - | - | 34.3 | 32.1 | 7 | 3 | 0.19 |
| Maruyama FFQ ^(69)^ | - | - | - | - | - | 26.4 | 34.4 | -23 | 3 | 0.53 |

| FFQ | Dairy | | | | | | | | | |
| --- | --- | --- | --- | --- | --- | --- | --- | --- | --- | --- |
|  | Total (g/d) | | | | | Milk (g/d) | | | | |
|  | Group level (mean or median) | | | | Correlation coefficient | Group level (mean or median) | | | | Correlation coefficient |
|  | Mean [median] (FFQ) | Mean [median] (DR) | Difference (%): FFQ−DR)/DR*100 | Statistics: 1: No difference 2: Difference 3: Not tested |  | Mean [median] (FFQ) | Mean [median] (DR) | Difference (%): FFQ−DR)/DR*100 | Statistics: 1: No difference 2: Difference 3: Not tested |  |
| 47-item FFQ ^(41)^ | - | - | - | - | - | 99.0 | 106.0 | -7 | 3 | 0.76 |
|  | - | - | - | - | - | 148.0 | 137.0 | 8 | 3 | 0.56 |
| DHQ ^(43)^ | 87.7 | 103.4 | -15 | 1 | 0.72 | - | - | - | - | - |
|  | 189.5 | 182.1 | 4 | 1 | 0.61 | - | - | - | - | - |
| BDHQ ^(43)^ | 96.4 | 103.4 | -7 | 1 | 0.60 |  |  |  |  |  |
|  | 180.7 | 182.1 | -1 | 1 | 0.54 |  |  |  |  |  |
| MDHQ ^(49)^ | 51 | 57 | -11 | 2 | 0.65 | - | - | - | - | - |
|  | 106 | 104 | 2 | 1 | 0.61 | - | - | - | - | - |
| FFQ in JACC ^(52)^ | - | - | - | - | - | 121.8 | 110.9 | 10 | 3 | 0.72 |
|  | - | - | - | - | - | 149.1 | 135.3 | 10 | 3 | 0.65 |
| JPHC FFQ at baseline ^(55)^ | 249.6 | 122.1 | 104 | 3 | 0.46 | - | - | - | - | - |
|  | 291.5 | 149.7 | 95 | 3 | 0.46 | - | - | - | - | - |
| JPHC_5y ^(57)^ | 194.0 | 124.0 | 57 | 3 | 0.52 | - | - | - | - | - |
|  | 207.0 | 152.0 | 37 | 3 | 0.64 | - | - | - | - | - |
| FFQ in JPHC-NEXT ^(65)^ | 200.0 | 105.0 | 90 | 3 | 0.58 | - | - | - | - | - |
|  | 314.0 | 147.0 | 114 | 3 | 0.52 | - | - | - | - | - |
| Short-FFQ in JPHC-NEXT ^(65)^ | 100.0 | 108.0 | -7 | 3 | 0.59 | - | - | - | - | - |
|  | 113.0 | 148.0 | -23 | 3 | 0.73 | - | - | - | - | - |
|  |  |  |  |  |  |  |  |  |  |  |
| Short version FFQ ^(68)^ | 111.1 | 76.4 | 45 | 3 | 0.55 | - | - | - | - | - |
| Maruyama FFQ ^(69)^ | 227.0 | 172.8 | 31 | 3 | 0.67 | - | - | - | - | - |

| FFQ | Fats and oils | | | | | Confectioneries | | | | |
| --- | --- | --- | --- | --- | --- | --- | --- | --- | --- | --- |
|  | Total (g/d) | | | | | Total (g/d) | | | | |
|  | Group level (mean or median) | | | | Correlation coefficient | Group level (mean or median) | | | | Correlation coefficient |
|  | Mean [median] (FFQ) | Mean [median] (DR) | Difference (%): FFQ−DR)/DR*100 | Statistics: 1: No difference 2: Difference 3: Not tested |  | Mean [median] (FFQ) | Mean [median] (DR) | Difference (%): FFQ−DR)/DR*100 | Statistics: 1: No difference 2: Difference 3: Not tested |  |
| 47-item FFQ ^(41)^ | - | - | - | - | - | 20.0 | 27.0 | -26 | 3 | 0.55 |
|  | - | - | - | - | - | 20.0 | 37.0 | -46 | 3 | 0.25 |
| DHQ ^(43)^ | 22.9 | 19.3 | 19 | 2 | 0.43 | 46.7 | 32.2 | 45 | 2 | 0.37 |
|  | 27.3 | 22.1 | 24 | 2 | 0.35 | 68.7 | 61.4 | 12 | 1 | 0.28 |
| BDHQ ^(43)^ |  |  |  |  |  | 44.0 | 32.2 | 37 | 2 | 0.33 |
|  |  |  |  |  |  | 68.2 | 61.4 | 11 | 2 | 0.52 |
| MDHQ ^(49)^ | - | - | - | - | - | 49 | 24 | 104 | 2 | 0.27 |
|  | - | - | - | - | - | 59 | 25 | 136 | 2 | 0.43 |
| FFQ in JACC ^(52)^ | - | - | - | - | - | 17.0 | 21.2 | -20 | 3 | 0.58 |
|  | - | - | - | - | - | 20.0 | 39.2 | -49 | 3 | 0.27 |
| JPHC FFQ at baseline ^(55)^ | 1.0 | 11.3 | -91 | 3 | 0.17 | - | - | - | - | - |
|  | 1.1 | 9.7 | -89 | 3 | 0.16 | - | - | - | - | - |
| JPHC_5y ^(57)^ | 14.0 | 11.0 | 23 | 3 | 0.24 | 17.0 | 28.0 | -41 | 3 | 0.48 |
|  | 14.0 | 10.0 | 48 | 3 | 0.21 | 27.0 | 49.0 | -45 | 3 | 0.38 |
| FFQ in JPHC-NEXT ^(65)^ | 12.0 | 12.0 | 1 | 3 | 0.30 | 18.0 | 31.0 | -42 | 3 | 0.61 |
|  | 14.0 | 10.0 | 44 | 3 | 0.06 | 28.0 | 46.0 | -39 | 3 | 0.48 |
| Short-FFQ in JPHC-NEXT ^(65)^ | 8.0 | 12.0 | -35 | 3 | 0.16 | 8.0 | 32.0 | -75 | 3 | 0.50 |
|  | 8.0 | 10.0 | -17 | 3 | -0.21 | 12.0 | 46.0 | -74 | 3 | 0.26 |
|  |  |  |  |  |  |  |  |  |  |  |
| Short version FFQ ^(68)^ | 10.8 | 9.0 | 20 | 3 | 0.17 | 40.6 | 21.6 | 88 | 3 | 0.24 |
| Maruyama FFQ ^(69)^ | - | - | - | - | - | 46.2 | 35.9 | 29 | 3 | 0.66 |

| FFQ | Beverages | | | | | | | | | |
| --- | --- | --- | --- | --- | --- | --- | --- | --- | --- | --- |
|  | Total (g/d) | | | | | Soft drinks (g/d) | | | | |
|  | Group level (mean or median) | | | | Correlation coefficient | Group level (mean or median) | | | | Correlation coefficient |
|  | Mean [median] (FFQ) | Mean [median] (DR) | Difference (%): FFQ−DR)/DR*100 | Statistics: 1: No difference 2: Difference 3: Not tested |  | Mean [median] (FFQ) | Mean [median] (DR) | Difference (%): FFQ−DR)/DR*100 | Statistics: 1: No difference 2: Difference 3: Not tested |  |
| 47-item FFQ ^(41)^ | - | - | - | - | - | - | - | - | - | - |
|  | - | - | - | - | - | - | - | - | - | - |
| DHQ ^(43)^ | - | - | - | - | - | 20.8 | 20.9 | 0 | 1 | 0.39 |
|  | - | - | - | - | - | 12.9 | 19.8 | -35 | 1 | 0.28 |
| BDHQ ^(43)^ | - | - | - | - | - | 18.1 | 20.9 | -13 | 1 | 0.46 |
|  | - | - | - | - | - | 0 | 19.8 | N/A | 2 | 0.32 |
| MDHQ ^(49)^ | - | - | - | - | - | 0 | 0 | 0.0 | 1 | 0.36 |
|  | - | - | - | - | - | 8 | 0 | 0.0 | 1 | 0.48 |
| FFQ in JACC ^(52)^ | - | - | - | - | - | - | - | - | - | - |
|  | - | - | - | - | - | - | - | - | - | - |
| JPHC FFQ at baseline ^(55)^ | - | - | - | - | - | - | - | - | - | - |
|  | - | - | - | - | - | - | - | - | - | - |
| JPHC_5y ^(57)^ | - | - | - | - | - | - | - | - | - | - |
|  | - | - | - | - | - | - | - | - | - | - |
| FFQ in JPHC-NEXT ^(65)^ | - | - | - | - | - | - | - | - | - | - |
|  | - | - | - | - | - | - | - | - | - | - |
| Short-FFQ in JPHC-NEXT ^(65)^ | - | - | - | - | - | - | - | - | - | - |
|  | - | - | - | - | - | - | - | - | - | - |
|  |  |  |  |  |  |  |  |  |  |  |
| Short version FFQ ^(68)^ | 805.9 | 619.3 | 30 | 3 | 0.38 | - | - | - | - | - |
| Maruyama FFQ ^(69)^ | - | - | - | - | - | - | - | - | - | - |

| FFQ | Seasonings and spices | | | | |
| --- | --- | --- | --- | --- | --- |
|  | Total (g/d) | | | | |
|  | Group level (mean or median) | | | | Correlation coefficient |
|  | Mean [median] (FFQ) | Mean [median] (DR) | Difference (%): FFQ−DR)/DR*100 | Statistics: 1: No difference 2: Difference 3: Not tested |  |
| 47-item FFQ ^(41)^ | - | - | - | - | - |
|  | - | - | - | - | - |
| DHQ ^(43)^ | - | - | - | - | - |
|  | - | - | - | - | - |
| BDHQ ^(43)^ | - | - | - | - | - |
|  | - | - | - | - | - |
| MDHQ ^(49)^ | - | - | - | - | - |
|  | - | - | - | - | - |
| FFQ in JACC ^(52)^ | - | - | - | - | - |
|  | - | - | - | - | - |
| JPHC FFQ at baseline ^(55)^ | 2.4 | 35.9 | -93 | 3 | 0.08 |
|  | 2.4 | 32.9 | -93 | 3 | 0.08 |
| JPHC_5y ^(57)^ | 4.0 | 36.0 | -88 | 3 | 0.12 |
|  | 5.0 | 33.0 | -85 | 3 | 0.12 |
| FFQ in JPHC-NEXT ^(65)^ | 22.0 | 138.0 | -84 | 3 | 0.25 |
|  | 23.0 | 114.0 | -80 | 3 | 0.16 |
| Short-FFQ in JPHC-NEXT ^(65)^ | 16.0 | 137.0 | -88 | 3 | 0.41 |
|  | 15.0 | 113.0 | -87 | 3 | 0.16 |
|  |  |  |  |  |  |
| Short version FFQ ^(68)^ | 62.2 | 93.2 | -33 | 3 | 0.19 |
| Maruyama FFQ ^(69)^ | - | - | - | - | - |

FFQ, Food frequency questionnaire; DR, Dietary record; 47-item FFQ, 47-item short food frequency questionnaire;

DHQ, Self-administered diet history questionnaire; BDHQ, Brief-type self-administered diet history questionnaire;

MDHQ, Meal-based Diet History Questionnaire; JPHC FFQ at baseline, 44-item food frequency questionnaire;

JPHC_5y, JPHC FFQ at 5-year follow-up; FFQ in JPHC-NeXT, Long-FFQ in JPHC-NeXT;

Short-FFQ in JPHC-NeXT, 66-item food frequency questionnaire for the Japan Public Health Centre-based

prospective Study for the Next Generation (JPHC-NEXT) follow-up survey; Short version FFQ,

Short version of the Shizuoka Prefecture version of the Food Intake Frequency Questionnaire; Maruyama FFQ,

Maruyama food frequency questionnaire.

Supplementary Table 4. The results of the validity assessment of dietary questionnaires among Japanese adults in additional papers

| FFQ | Participant characteristics | | | | Statistics: Correlations  1: Correlation 2: Adjusted correlation (energy) 3: Disattenuated or intraclass correlation | Type of correlation coefficient P: Pearson S: Spearman | Nutrients | | | | | Food groups | | | | |  |
| --- | --- | --- | --- | --- | --- | --- | --- | --- | --- | --- | --- | --- | --- | --- | --- | --- | --- |
|  |  |  |  |  |  |  | Correlations | | | | | Correlations | | | | |  |
|  | Sex M: Male F: Female | Age | n | Other characteristics |  |  | Mean [median]^‡^ | Low | | High | | Mean [median]^‡^ | Low | | High | | Note |
|  |  |  |  |  |  |  |  | Nutrient | Value | Nutrient | Value |  | Food | Value | Food | Value |  |
| 47-item FFQ ^(39)^ | M | 40-69 | 29 | Living in Amami | 3 | P | 0.38 | Saturated fatty acid | 0.23 | Vitamin D | 0.64 | - | - | - | - | - |  |
|  | F | 58.3(40-69) | 37 | Living in Amami | 3 | P | 0.51 | Vitamin C | 0.25 | Saturated fatty acid | 0.81 | - | - | - | - | - |  |
| 47-item FFQ ^(40)^ | M | 72.5 (65-85) | 78 | Elderly | 1 | S | 0.24 | n -6 polyunsaturated fatty acid | -0.02 | Calcium | 0.49 | - | - | - | - | - |  |
|  | F | 73.8 (66-88) | 65 | Elderly | 1 | S | 0.24 | n -6 polyunsaturated fatty acid | 0.01 | Vitamin B_2_ | 0.4 | - | - | - | - | - |  |
| DHQ ^(42)^ | F | 38-69 | 47 | Mild hypercholesterolemia | 3 | P | 0.48 | Niacin | 0.19 | Saturated fatty acid | 0.75 | - | - | - | - | - |  |
| DHQ ^(45)^ | M | 32-76 | 92 | Married couple | 2 | S | 0.54 | Maltose | 0.33 | Lactose | 0.67 | - | - | - | - | - | Not classified in NHNS food groups because only sugar is evaluated. |
|  | F | 31-69 | 92 | Married couple | 2 | S | 0.49 | Maltose | 0.23 | Lactose | 0.65 | - | - | - | - | - | Not classified in NHNS food groups because only sugar is evaluated. |
| **B**DHQ ^(45)^ | M | 32-76 | 92 | Married couple | 2 | S | 0.42 | Galactose | 0.06 | Starch | 0.62 | - | - | - | - | - | Not classified in NHNS food groups because only sugar is evaluated. |
|  | F | 31-69 | 92 | Married couple | 2 | S | 0.44 | Galactose | -0.01 | Sucrose Lactose | 0.54 | - | - | - | - | - | Not classified in NHNS food groups because only sugar is evaluated. |
| BDHQ ^(46)^ | M | 40-69 | 343 | - | 0 | S | 0.27 | Vitamin A | 0.07 | Carbohydrate | 0.45 | 0.29 | Potato | 0.07 | Dairy | 0.57 |  |
|  | F | 40-69 | 343 | - | 0 | S | 0.26 | Sodium | 0.12 | Folate Calcium | 0.36 | 0.27 | Sugars | 0.08 | Dairy | 0.49 |  |
| BDHQ ^(47)^ | M/F | 82-94 | men 36, women 44 | Very old Japanes | 2 | S | 0.40 | Vitamin B_1_ | 0.22 | Vitamin B_6_ | 0.64 | 0.47 | Sugars | 0.05 | Dairy | 0.55 |  |
| BDHQ3y ^(48)^ | M/F | 3-4 | 61 | Childrem | 2 | S | 0.27 | Vitamin B_1_ | -0.13 | Cholesterol | 0.51 | 0.37 | Potato | -0.1 | Dairy | 0.56 |  |
| FFQ in JACC^(53)^ | M/F | 20-79 | 85 | - | 2 | - | 0.31 | n -6 polyunsaturated fatty acid | 0.16 | Animal fat | 0.51 | - | - | - | - | - |  |
| JPHC FFQ at baseline ^(54)^ | M | - | 207 | JPHCvalidation study | 3 | P | 0.26 | Protein | -0.04 | Cabohydrate | 0.58 | - | - | - | - | - | JPHC-modified 36 item FFQ; based on food composition method; main objective was to predict nutrient intake |
|  | F | - | 166 | JPHCvalidation study | 3 | P | 0.38 | Niacin | 0.18 | Calcium | 0.67 | - | - | - | - | - | JPHC-modified 36 item FFQ; based on food composition method; main objective was to predict nutrient intake |
| JPHC_5y ^(58)^ | M | mean 55.6 | 102 | JPHC-cohort I | 2 | S | 0.34 | - | - | - | - | - | - | - | - | - | Evaluated only for selenium |
|  | F | mean 54.6 | 113 | JPHC-cohort I | 2 | S | 0.26 | - | - | - | - | - | - | - | - | - | Evaluated only for selenium |
| JPHC_5y ^(59)^ | M | mean 55.6 | 102 | JPHC-cohort I | 2 | S | 0.43 | Dietary fibre, insoluble & Dietary fibre, total | 0.43 | Dietary fibre, soluble | 0.44 | 0.37 | Vegetables | 0.18 | Grains | 0.49 | Evaluated only for dietary fibre and main food sources |
|  | F | mean 54.6 | 113 | JPHC-cohort I | 2 | S | 0.39 | Dietary fibre, soluble | 0.36 | Dietary fibre, insoluble & dietary fibre, total | 0.4 | 0.22 | Vegetables | 0.15 | Other | 0.35 | Evaluated only for dietary fibre and main food sources Other: includes dietary fibre from all foods except for grains, vegetables, and fruit |
| JPHC_5y ^(60)^ | M | mean 58.9 | 174 | JPHC-cohort II | 2 | S | 0.49 | Vitamin B1 | 0.28 | Calcium | 0.65 | 0.41 | Seaweed | 0.11 | Dairy | 0.69 |  |
|  | F | mean 55.9 | 176 | JPHC-cohort II | 2 | S | 0.45 | Cholesterol | 0.18 | Calcium | 0.64 | 0.3 | Mushrooms | 0.12 | Dairy | 0.64 |  |

| JPHC_5y ^(61)^ | M | mean 55.6 | 102 | JPHC-cohort I | 3 | S | 0.57 | - | - | - | - | - | - | - | - | - | Evaluated only for folate |
| --- | --- | --- | --- | --- | --- | --- | --- | --- | --- | --- | --- | --- | --- | --- | --- | --- | --- |
|  | F | mean 54.6 | 113 | JPHC-cohort I | 3 | S | 0.47 | - | - | - | - | - | - | - | - | - | Evaluated only for folate |
|  | M | mean 58.9 | 174 | JPHC-cohort II | 3 | S | 0.63 | - | - | - | - | - | - | - | - | - | Evaluated only for folate |
|  | F | mean 55.9 | 176 | JPHC-cohort II | 3 | S | 0.63 | - | - | - | - | - | - | - | - | - | Evaluated only for folate |
| JPHC_5y ^(62)^ | M | mean 55.6 | 102 | JPHC-cohort I | 2 | S | 0.46 | Vitamin D | 0.26 | Pantothenic acid | 0.69 | - | - | - | - | - | - |
|  | M | mean 58.9 | 174 | JPHC-cohort II | 2 | S | 0.43 | Vitamin E | 0.24 | Copper | 0.60 | - | - | - | - | - | - |
|  | F | mean 54.6 | 113 | JPHC-cohort I | 2 | S | 0.41 | Retinol equivaltent | 0.31 | Copper | 0.58 | - | - | - | - | - | - |
|  | F | mean 55.9 | 176 | JPHC-cohort II | 2 | S | 0.43 | Vitamin B_12_ | 0.27 | Pantothenic acid | 0.61 | - | - | - | - | - | - |
| JPHC_5y ^(63)^ | M | 40-69 | 69 | Middle-Aged Urban Cancer Screenees | 3 | S | 0.57 | Vitamin A | 0.23 | Vitamin D | 0.88 | 0.51 | Seasonings and spices | 0.1 | Meats | 0.7 | - |
|  | F | 40-69 | 74 | Middle-Aged Urban Cancer Screenees | 3 | S | 0.47 | Niacin | 0.32 | Vitamin K | 0.94 | 0.51 | Seasonings and spices | −0.36 | Dairy | 0.76 | - |
| JPHC_5y ^(64)^ | M | mean 55.6 | 102 | JPHC-cohort I | 3 | S | 0.47 | - | - | - | - | - | - | - | - | - | Evaluated only for polyphenol |
|  | F | mean 54.6 | 113 | JPHC-cohort I | 3 | S | 0.37 | - | - | - | - | - | - | - | - | - | Evaluated only for polyphenol |
|  | M | mean 58.9 | 174 | JPHC-cohort II | 3 | S | 0.44 | - | - | - | - | - | - | - | - | - | Evaluated only for polyphenol |
|  | F | mean 55.9 | 176 | JPHC-cohort II | 3 | S | 0.5 | - | - | - | - | - | - | - | - | - | Evaluated only for polyphenol |
| FFQ in JPHC-NEXT ^(66)^ | M | - | 92 | Collegiate Athletes | 3 | S | 0.3 | Zinc | 0.1 | Calcium | 0.57 | 0.32 | Sugar | 0.17 | Dairy | 0.72 | - |
|  | F | - | 64 | Collegiate Athletes | 3 | S | 0.32 | Fat | -0.08 | Magnesium | 0.59 | 0.34 | Sugar | -0.11 | Dairy | 0.58 | - |
| FFQ in JPHC-NEXT ^(67)^ | M | 40-74 | 98 | JPHC-NEXT | 3 | S | 0.47 | Niacin | 0.25 | Carbohydrate | 0.74 | 0.48 | Seaweed | 0.09 | Grains | 0.7 | - |
|  | F | 40-74 | 139 | JPHC-NEXT | 3 | S | 0.46 | n -6 polyunsaturated fatty acid | 0.23 | Vitamin B12 | 0.61 | 0.44 | Fats and oils | 0.07 | pulses | 0.64 |  |
| FFQ, Food frequency questionnaire; DR, Dietary record; 47-item FFQ, 47-item short food frequency questionnaire; DHQ, Self-administered diet history questionnaire; BDHQ, Brief-type self-administered diet history questionnaire; MDHQ, Meal-based Diet History Questionnaire; JPHC FFQ at baseline, 44-item food frequency questionnaire; JPHC_5y, JPHC FFQ at 5-year follow-up; FFQ in JPHC-NeXT, Long-FFQ in JPHC-NeXT; Short-FFQ in JPHC-NeXT, 66-item food frequency questionnaire for the Japan Public Health Centre-based prospective Study for the Next Generation (JPHC-NEXT) follow-up survey; Short version FFQ, Short version of the Shizuoka Prefecture version of the Food Intake Frequency Questionnaire; Maruyama FFQ, Maruyama food frequency questionnaire. | | | | | | | | | | | | | | | | | |
